# The youth mental health crisis: Quasi-experimental evidence on the role of school closures

**DOI:** 10.1126/sciadv.adh4030

**Published:** 2023-08-18

**Authors:** Christina Felfe, Judith Saurer, Patrick Schneider, Judith Vornberger, Michael Erhart, Anne Kaman, Ulrike Ravens-Sieberer

**Affiliations:** ^1^ University of Konstanz, Konstanz, Germany.; ^2^ Center of Economic Performance, London, UK.; ^3^ CESifo, Munich, Germany.; ^4^ IZA, Bonn, Germany.; ^5^ University of Würzburg, Würzburg, Germany.; ^6^ Frankfurt Laboratory for Experimental Economic Research, Goethe University Frankfurt, 60323 Frankfurt, Germany.; ^7^ The Center for Leadership and Behavior in Organizations, Goethe University Frankfurt, 60323 Frankfurt, Germany.; ^8^ University Medical Center Hamburg-Eppendorf, Hamburg, Germany.; ^9^ Alice Salomon University of Applied Science, Berlin Apollon University of Applied Science, 28359 Bremen, Germany.

## Abstract

During the COVID-19 pandemic, the youth mental health crisis has reached unprecedented levels. To which extent school closures, one of the most heavily debated pandemic measures, have contributed to or even caused this crisis is largely unknown. We seek to narrow this blind spot, by combining quasi-experimental variation in school closure and reopening strategies across the German federal states at the onset of the pandemic with nationwide, population-based survey data on youth mental health and high-frequency data from the largest crisis helpline. We show that prolonged school closures led to a substantial deterioration in youth health-related quality of life, precipitating early signs of mental health problems. The effects were most severe among boys, younger adolescents, and families with limited living space. We further provide evidence that family problems are a major issue that adolescents were struggling with when denied access to school. Overall, school closures largely explain the deterioration of youth mental health over the first pandemic wave.

## Teaser

School closures cause the youth mental health problems during the COVID-19 pandemic.

## INTRODUCTION

The rapid spread of the coronavirus severe acute respiratory syndrome coronavirus 2 (SARS-CoV-2) and the associated respiratory disease coronavirus disease 2019 (COVID-19) motivated governments around the globe to impose marked policy measures such as physical distancing, contact reduction, working from home, or homeschooling. The pandemic and its related measures massively affected people’s life and left scars on people’s mental health (*1*–*7*). While the virus disproportionally affected the elderly, negative psychological consequences were particularly pronounced during childhood and adolescence (*1*, *3*, *4*, *8*, *9*), the most dynamic and thus vulnerable period in human life from the perspective of developmental psychology (*10*–*12*). Globally, child and adolescent mental health problems are at unprecedented levels. Recent studies report a doubling of child and adolescent anxiety and depression levels, compared with pre-pandemic estimates (*13*). Worldwide, at least 13% of people between the ages of 10 and 19 now live with a diagnosed mental health disorder (*14*). Despite these alarming numbers, studies on the role played by the different aspects of the pandemic, particularly its related measures, are rare and of correlational nature at best (*15*–*17*). Existing studies struggle with disentangling the consequences of the multiple pandemic measures and the pandemic itself and, as such, are of limited use for policy design. One exception is a recent study on Sweden documenting a reduction in mental health care demand among upper-secondary students who were moved to remote learning at the onset of the pandemic. This study relies on a credible comparison with students from lower levels who were granted access to schools throughout (*18*). Yet so far, it is unclear whether the documented reduction in health care demand reflects an actual change in mental health or hides an increasing number of unnoticed mental health problems and thus may convey a false all-clear signal.

We fill this void and shed light on the potential costs and risks of school closures by relying on (i) unique German survey data on adolescents’ mental health [collected between 26 May and 10 June 2020 and thus during the first pandemic wave (*19*), as well as between August 2015 and November 2017 and thus before the pandemic serving as a benchmark (*20*)] and (ii) high-frequency data from the largest German crisis helpline (available for January 2019 until December 2020). These data facilitate insights into adolescents’ (inner) life at times when the usual support and warning systems (e.g., teachers and pediatricians) were largely suspended. To isolate the overall strain imposed by the pandemic or by further pandemic measures, we exploit quasi-experimental variation in the length of school closures resulting from state-specific regulations for the different grade levels and school tracks. To leverage this variation and provide causal estimates for the mental health consequences of school closures (*21*), we processed all state-specific corona protection ordinances and compiled a dataset enabling us to assign each adolescent in our data the respective mandated weeks of school access restriction (*22*) and to estimate their causal effect on youth mental health.

To put things into perspective, school closures were among the first measures taken to fight the viral spread. By mid-April 2020, governments of 151 countries had mandated partial or full school closures, exempting more than 81% of all enrolled learners from in-person education (*23*). Schools are not only children’s and adolescents’ place to learn but also their place to engage and establish social interactions. For those in need, schools represent the first point of contact and guard their well-being (*2*, *4*). Thus, school closures imply a marked disruption to children’s and adolescents’ lives, during a phase of life when engaging, predictable environments and stable, positive social interactions are crucial for promoting children’s and adolescents’ socio-emotional development and preventing challenging behaviors (*24*). Moreover, school closures together with the remaining pandemic measures result in an unprecedented intersection of work, school, and home life, putting parents under enormous pressure and thus limiting, if not even disabling, their ability to buffer the detrimental consequences for their children (*15*, *25*, *26*).

### The German federal system as a natural laboratory

To disentangle the overall impact and single out the consequence of school closures and thus of one specific pandemic measure, we zoom in to one country, the Federal Republic of Germany. The German federal states enjoy cultural and educational sovereignty and thus assume responsibility for their school system. Accordingly, each state independently decided about the school closure and school reopening strategy resembling a natural laboratory allowing for causal identification of the impact of the length of school closures on youth mental health.

To provide more context, in reaction to the rapid spread of the COVID-19 virus, all 16 German federal governments mandated statewide school closures between 16 and 18 March 2020. From 20 April 2020, onward, the states reopened schools gradually, but each state followed its own strategy. In general, priority was given to graduating cohorts [corresponding to grade level 4 and in two states to grade level 6, for primary school, and, depending on the school track and state, to grade level 9, 10, 12, or 13 for secondary school; see also (*27*) for further details on the German school system]. The return of the remaining grade levels was organized stepwise and varied across states. Some states followed a strict chronological order drawing in first the graduating cohorts followed then successively by the younger ones, while others gave priority to both graduating and entry-level cohorts followed by the intermediate ones. Thus, the number of weeks an individual was exempted from in-person learning depended on his/her state of residence, the grade level he/she belonged to, and the school track he/she attended (*22*). Any further state-specific variation, such as variation in the overall course of the pandemic or in further pandemic measures applied equally to all cohorts, and is thus orthogonal to the variation in the weeks of school closure leveraged within this study (see table S2A for a state-level comparison of cases and deaths and table S3 for a state-level comparison of the stringency of further pandemic measures; for an international comparison of cases, deaths, and overall stringency of pandemic measures, please refer to table S2B).

**Fig. 1.**
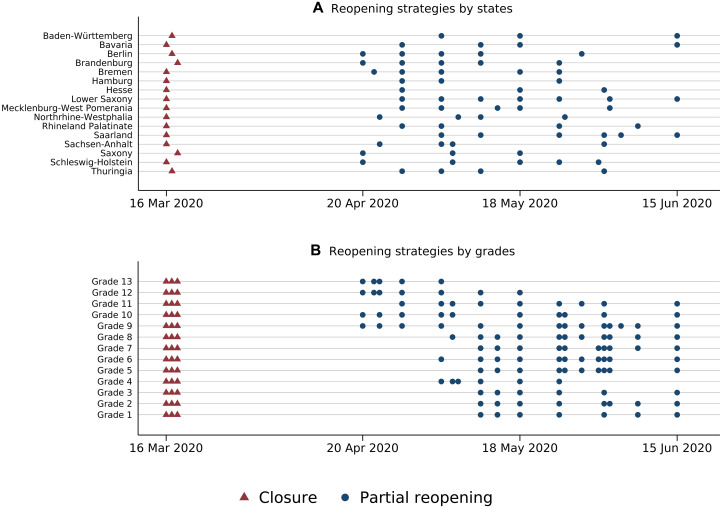
Variation in school closure and reopening strategies. Data are compiled on the basis of a comprehensive screening of the state-specific corona protection ordinances (*22*). Each blue dot represents the date when the respective federal government (partially) reopened schools for selected grade levels, possibly restricted to the grade levels of certain school tracks (**A**) and the resulting variation in the reopening dates by grade levels across the federal states and possibly school tracks (**B**). As shown in fig. S1, substantial variation remains when displaying the variation within the different school tracks separately.

In the absence of a comparative and transparent overview of the length of school closures, we processed all state-specific corona protection ordinances and compiled a dataset on the state-specific school closure and reopening strategies (*22*). Figure 1A illustrates the strategies adopted by the 16 German federal states, differing in the timing, the intervals, and the overall duration of the reopening phase. Figure 1B displays the resulting variation by grade levels (subsuming the variation across school tracks; see fig. S1 for the variation by school track). Two facts stand out: (i) the priority given to the graduating students who started to return already after 4.7 weeks and (ii) the substantial variation within grade levels (ranging from 4.7 to 13 weeks).

### Identifying the mental health impact of school closures

We exploit the described variation in the school closure and reopening strategies to identify the mental health consequences of prolonged school closures. More precisely, we use a two-way fixed-effects approach (please refer to Materials and Methods for an example illustration of this approach) and rely on the within-state and within–grade level (differentiated by school track) variation in the length of school closures (*21*, *28*, *29*). Specifically, we first compare the mental health of adolescents residing in the same state but attending different grade levels (and, possibly, different school tracks). This comparison isolates any state-specific level differences of adolescents’ mental health, subsuming both any pre-pandemic differences in adolescents’ mental health (see also fig. S2A) and any variation in adolescents’ mental health because of state-specific differences in the severity of the pandemic or the stringency of the pandemic measures. Second, we compare the mental health of adolescents attending the same grade level (and school track) but residing in different federal states. This comparison isolates any age-specific differences in mental health (see also fig. S2B) as well as differences in mental health that may arise because adolescents attending different grade levels or school tracks may cope or struggle differently with the pandemic and its related measures. The two-way fixed-effects approach then captures any differences arising due to the remaining within-state and within–grade level (and school track) variation in the length of school closures. To interpret the resulting estimate as the causal effects of prolonged school closures, the only necessary assumption is that neither the pandemic severity nor further pandemic measures vary at this level (within states across grade levels or school tracks). This assumption is plausible as (i) case rates among adolescents were negligible (at least in the first wave of the pandemic) and case rates and deaths of parents or grandparents should be comparable across the age ranges of the children in our sample; and (ii) there are no further pandemic measures targeting explicitly specific age groups, grade levels, or school tracks (*30*). To further probe this assumption, we test the robustness of our results when considering state-level differences in the severity of the pandemic and in the stringency of further pandemic measures.

### Nationwide data on youth mental health

To get at youth mental health beyond the statistics based on the realized health care demand, we make use of unique German survey data on adolescents’ mental health, the so-called BELLA (short for Behavior and Wellbeing of Children and Adolescents in Germany) cohort study. BELLA forms part of the nationwide, representative German National Health Interview and Examination Survey for Children and Adolescents (*20*, *31*–*33*). We rely on the COVID-19 online issue of the survey conducted with 1040 11- to 17-year-olds between 26 May and 10 June 2020, also referred to as the COvid-19 and PSYchological health survey [short COPSY; (*19*, *34*)]. In addition, we have data on the parents of all 11- to 17-year-old adolescents participating in COPSY. To quantify the overall deterioration of youth mental health during the first pandemic wave and to determine the role of school closures therein, we also consider the last pre-pandemic wave of BELLA collected in person between August 2015 and November 2017 (*n* = 1556 11- to 17-year-olds). We use sampling weights to respect the population structure and scale our results to make representative and policy-relevant statements (*19*, *34*).

Merging COPSY with the data on school closures (by state of residence, grade level, and school track) results in a sample of *n* = 907 11- to 17-year-old adolescents, attending grade levels 4 to 13 in spring 2020 (see table S4 for the sample summary statistics). Restricting the pre-pandemic wave of BELLA to children attending those grade levels and with information about their state of residence, the resulting pre-pandemic sample comprises *n* = 1334 11- to 17-year-old adolescents (see table S5 for the respective sample summary statistics). The length of school closures varies between 4.7 and 10.1 weeks for the adolescents in our sample (we top code the weeks of school closure with the beginning of the survey on 26 May 2020, as we lack the exact survey date for each participant). The data allow for a snapshot of youth mental health toward the end of the first lockdown and, as such, for an analysis of the short-run mental health effects of prolonged school closures (4.0 additional weeks on average, considering that each adolescent was exempted from school for at least 4.7 weeks). It includes internationally established and validated instruments for measuring adolescents’ health-related quality of life (HRQoL) using the KIDSCREEN-10 Index (*35*) and screening instruments for mental health problems, such as the HBSC (short for Health Behavior in School-aged Children) Symptom Checklist (HBSC-SCL) to check for psychosomatic complaints (*36*), the Strengths and Difficulties Questionnaire (SDQ) to elicit behavioral and emotional difficulties (*37*), the Center for Epidemiological Studies Depression Scale for Children (CES-DC) (*38*), and the Screen for Child Anxiety Related Emotional Disorders (SCARED) (*39*) scale to measure levels of depressive and anxiety symptoms, respectively. In addition, it provides measures for parents’ mental health, relying on the Patient Health Questionnaire (PHQ-8) (*40*), and parental reports on the family climate (FC) (*41*).

To get more detailed insights into youth concerns and worries, we further draw upon high- frequency, real-time data from the largest and most frequented crisis helpline for children and adolescents in Germany (“Nummer gegen Kummer,” particularly the “Kinder- und Jugendtelefon”). We rely on data on all calls entering this crisis helpline between 1 January 2019 and 31 December 2020, the topics discussed during the calls, and the basic sociodemographics of the calling party (including sex, age, and federal state from where the call originated). Merging those data with the data on school closures (by state and age as a grade level proxy) results in a sample of *n* = 126,006 calls of 11- to 17-year-old adolescents, of which 51,833 are informative about the reasons adolescents are calling (the remaining calls relate mainly to unspecified psychosocial and health issues; for more details, see table S6).

## RESULTS

### School closures cause mental health issues in the short run

**Fig. 2.**
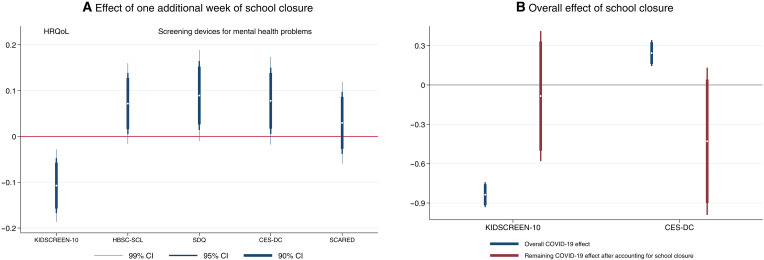
Results. (**A**) The short-run impact of school closure on HRQoL and several screening devices for mental health problems (including the 99, 95, and 90% confidence interval) for the effect of one additional week of school closure on standardized measures (mean, 0; SD, 1) of adolescents’ self-reported HRQoL (KIDSCREEN-10), and screening instruments for mental health problems ( HBSC-SCL, SDQ, CES-DC, and SCARED). For all scales, except KIDSCREEN-10, higher values express more health problems. For exact values, see table S7A. Each bar results from a separate two-way fixed-effects regression (controlling for a set of state and school track–specific grade level fixed effects as well as for age and sex) using ordinary least squares with sampling weights and SEs clustered at the state*grade level*school track (see Eq. 1). (**B**) The decline in HRQoL (higher values express less health problems) and depressive symptoms (CES-DC, higher values express more health problems) (including the 95 and 90% confidence interval) between 2017 and 2020. Each bar represents a separate estimation, with the first estimation in each panel not accounting for school closure and the second estimation including it. All estimations control for age and sex and use ordinary least squares with sampling weights and SEs that are clustered at the state*school track *grade level (as detailed in Eqs. 2 and 3). For detailed regression results, see table S7 (B and C).

The effects of school closures on adolescents’ HRQoL and mental health symptoms are summarized in Fig. 2A (see also table S7A). The results show that each additional week of school closure is associated with a decrease in HRQoL (0.107 SD, *P <* 0.001) and increases in psychosomatic symptoms (0.072 SD, *P <* 0.05), behavioral and emotional health problems (0.089 SD, *P <* 0.05), and depressive symptoms (0.079 SD, *P <* 0.05). For the time period under study, there are no effects on anxiety symptoms.

These results are robust to a battery of sensitivity checks shown in table S8. To discern one of the most pressing issues, namely, that our results are driven by state differences in pandemic severity or other pandemic measures, we estimate a series of alternative regression models controlling additionally for state-level pandemic severity and state-level stringency of a series of further pandemic measures (see also Materials and Methods for a detailed description of the underlying method and table S9 for the estimation results). Reassuringly, the estimated effect of the weeks of school closure remains unchanged throughout. Moreover, only 1 of the 65 coefficients estimating a possible direct impact of further pandemic measure on youth mental health is significant at the 5% level (the impact of restricting outdoor activities on anxiety).

To further dispel the concern that our results are driven by preexisting level differences in adolescents’ mental health, we additionally draw upon the pre-pandemic wave of the BELLA cohort study providing us with measures for two of the four mental health measures, specifically KIDSCREEN-10 and CES-DC (see also Materials and Methods for a detailed description of the underlying method and table S7C for the estimation results). These additional analyses yield the following crucial results: (i) there is no significant correlation between the pre-pandemic level of adolescents’ mental health and the mandated weeks of school closures (HRQoL: −0.001 SD, *P* = 0.976; CES-DC: −0.016 SD, *P* = 0.522); and (ii) the estimated effects of the weeks of school closure during the pandemic on adolescents’ mental health barely alter (in comparison to the respective baseline effects, shown in table S7A) when accounting for possible pre-pandemic level differences: The effect on HRQoL corresponds to −0.086 SD (in comparison to −0.107 SD at baseline) and on depressive symptoms to 0.076 SD (in comparison to 0.079 SD at baseline).

We further make use of the pre-pandemic wave of the BELLA cohort study and compare it to COPSY to describe the overall deterioration in adolescents’ mental health over the first pandemic wave. As shown in Fig. 2B (blue bars), HRQoL declined by 0.824 SD (*P <* 0.001) and depressive symptoms increased by 0.246 SD (*P <* 0.001) over the period of the first lockdown (see also table S7B), corroborating previous results (*19*, *42*, *43*). However, as soon as we consider the impact of school closures, these estimates loose magnitude and are no longer distinguishable from zero [see Fig. 2B (red bars) and table S7C]. In other words, we cannot reject the hypothesis that the mandated school closures explain the entire deterioration of adolescents’ mental health. However, one should interpret these results with caution: The respective 95% confidence intervals are rather large and allow for the possibility that the prolonged school closures explain only 31.1% of the decrease in HRQoL and 17.2% of the increase in depressive symptoms.

### Not everyone suffered equally

**Fig. 3.**
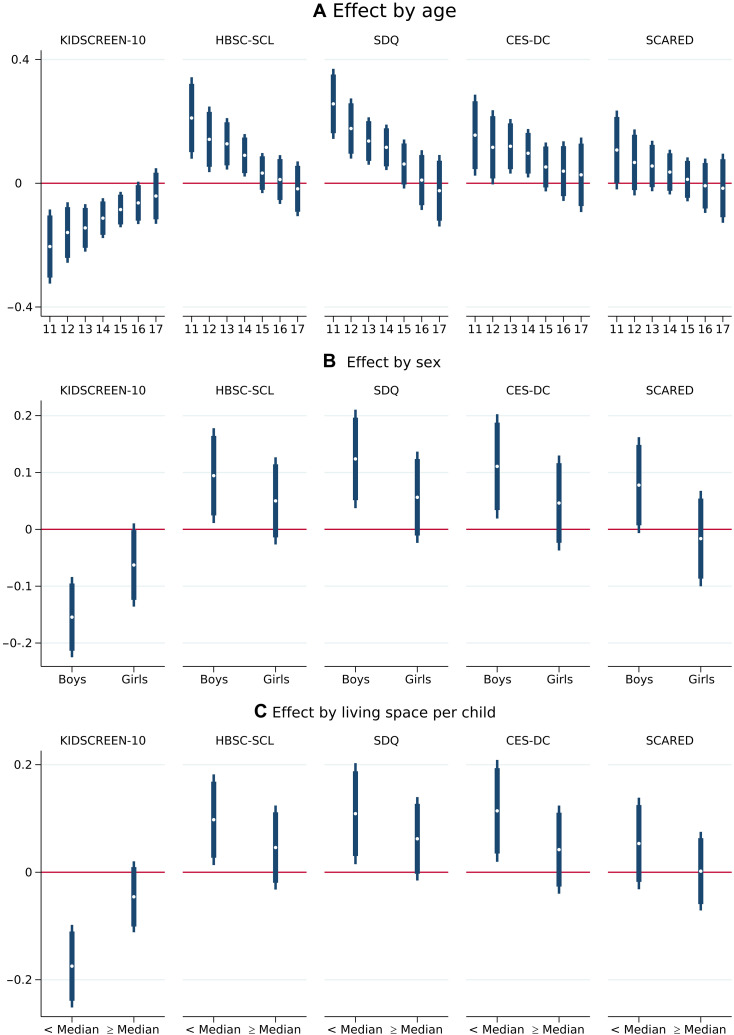
Results by sociodemographic characteristics. The short-run impact of school closure on HRQoL and screening devices for mental health (including the 95 and 90% confidence interval) for the effect of one additional week of school closure on standardized measures (mean, 0; SD, 1) of adolescents’ self-reported HRQoL (KIDSCREEN-10), and screening instruments for mental health problems (HBSC-SCL, SDQ, CES-DC, and SCARED) separately by age (**A**), sex (**B**), and living space per child (**C**). For all scales, except KIDSCREEN-10, higher values express more health problems. For more details see Eqs. 5 to 7 and table S10 for the detailed regression results.

The strain and the ability to shoulder the burden imposed by school closures likely varied by age, sex, and living conditions (see Fig. 3 and table S10). Subgroup analysis reveals that younger children struggled most with the strain caused by school closures, with the effects declining monotonically with age (Fig. 3A). The youngest in our sample, the 11-year-olds, experienced marked losses in HRQoL (−0.205 SD, *P <* 0.001) and an increase in psychosomatic symptoms (0.211 SD, *P <* 0.001) as well as in behavioral and emotional problems (0.257 SD, *P <* 0.001) and in depressive symptoms (0.157 SD, *P <* 0.05). Effects quickly fade out, loosing precision by mid-adolescence.

Boys coped much worse with school closures than girls (Fig. 3B). This is visible in a significant drop in HRQoL (−0.154 SD, *P <* 0.001) and an increase in psychosomatic symptoms (0.094 SD, *P <* 0.05), behavioral and emotional problems (0.124 SD, *P <* 0.001), and depressive symptoms (0.112 SD, *P <* 0.05).

To proxy the living conditions at home, we analyze the effects separately by the living space available per school-aged child (Fig. 3C). In homes with limited living space (below the median), adolescents suffered most from the burden imposed by the school closure, visible in a deterioration of their HRQoL (KIDSCREEN-10: −0.175 SD, *P <* 0.001) and an increase in symptoms of mental health problems (HBSC-SCL: 0.098 SD, *P <* 0.05; SDQ: 0.109 SD, *P <* 0.05; CES-DC: 0.115 SD, *P <* 0.05).

### Family problems—A major issue during school closures

The results on the consequences of prolonged school closures on youth self-reported mental health are alarming. Families were largely left alone to deal with the unprecedented situation at home, including the multiple burden of juggling work, school, and family life simultaneously. Using parental reports available in COPSY reveals that prolonged school closures had a detrimental effect on the FC (−0.14 SD, *P <* 0.001; using Eq. 1; see also table S11A, column 1). Analyzing subgroups, we find the same pattern in FC as in youth mental health (see table S11, B to D, column 1): Effects are strongest in families with younger children (−0.234 SD, *P <* 0.001), with boys (−0.169 SD, *P <* 0.001), and with limited living space (−0.203 SD, *P <* 0.001). However, we cannot detect any direct effect of prolonged school closures on available measures of parents’ mental health (table S11, column 2).

For the design of preventive or remediation measures, more detailed knowledge of the issues adolescents struggling with is needed. For this purpose, we draw upon high-frequency, real-time data from the largest and most frequented crisis helpline for children and adolescents in Germany (Nummer gegen Kummer, particularly the Kinder- und Jugendtelefon).

**Fig. 4.**
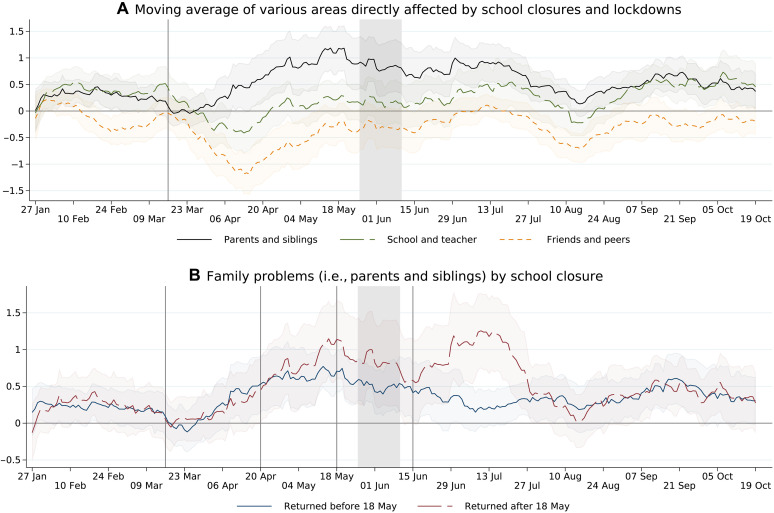
Changes in crisis helpline call volume in 2020. (**A**) Twenty-eight–day moving averages of call volumes. Call volume is the residuals of a regression in which the number of calls times their average duration is regressed on a set of month and weekend dummies. Residuals are standardized to have mean of 0 and SD of 1 for 2019. The shaded area indicates the 95% confidence interval. (**B**) The same moving average as in (A) but adolescents returning to school at or before 18 May and adolescents returning after 18 May, respectively. The vertical lines correspond to the date of nationwide school closure (16 to 18 March 2020), and the respective dates of school reopening for the early returners (from 20 April 2020 onward) and late returners (from 18 May 2020 until 15 June 2020). The gray area indicates the time when COPSY was conducted. For detailed regression results, see the table S12.

To start, we describe the overall volume change in calls grouped into three categories: calls due to family problems, problems with friends and peers, and problems related to school and teachers. Figure 4A displays the relative change in call volumes measured in SD over the course of 2020 relative to 2019. The immediate, large, and persistent increase in calls because of family problems catches the eye. In contrast, calls because of school problems remained unchanged, and calls because of problems with friends and peers even declined. To relate these changes with the school closures, Fig. 4B shows the development separately for adolescents below and above the median of the weeks of school closures (those returning before and after 18 May to school). Notable, there is a parallel, marked increase in the call volume related to family problems for both groups during the first phase of the pandemic when schools were closed for everyone. However, as soon as schools started opening their doors by the end of April 2020 for some of the students, the upward movement of the calls related to family problems stopped and turned into a downward movement ending up only slightly above pre-pandemic levels by summer 2020. In contrast, for those barred from returning to school until 18 May or longer, the upward trend continued at high levels and increased even further to peak by mid-July 2020 to plummet only by the end of July when summer remediation programs took off. Those descriptive results are confirmed by an autoregressive moving average estimation taking the autocorrelation structure of the helpline calls into account (see Materials and Methods for the underlying method and fig. S3).

## DISCUSSION

We relied on quasi-experimental variation in school reopening strategies across the German federal states (by grade level and school track) to identify the impact of school closures on youth mental health. Drawing upon nationwide, population-based survey data on adolescents’ mental health before and during the COVID-19 pandemic allowed us to determine the mental health impact of the pandemic and particularly of one of the most heavily debated pandemic measures on youth mental health. We showed that school closures substantially contributed to the recent aggravation in youth mental health problems. Using data from crisis helplines allowed us to shed light on the problems preoccupying adolescents during the pandemic, mostly struggling with family problems. Noteworthy, adolescents exposed to longer school closures struggled with these problems more and for a prolonged time.

Our results likely reflect only the tip of the iceberg. We only provide insights into the short-run effects of prolonged school closures on youth mental health. In addition, our estimates correspond to the effects of initial school closures only, lasting between 4.7 and 10.1 weeks. In Germany, 25 or more additional weeks of school closure followed; in other countries, such as the United States, students were exempted from in-person learning for 71 weeks in total (*23*). The effects may obviously not accumulate week by week. Many adolescents may have adjusted to and learned to live with the different situation. However, the pandemic and its related measures represented an immense burden for adolescents and their families, depriving adolescents from the positive social interactions urgently needed for a swift recovery to happen.

Having said this, there is immense variety in the severity of the pandemic and the stringency of the pandemic measures across countries (see table S2B). Whether our results hold in these different contexts is a priori unclear and requires additional cross-country analyses.

Moreover, to what extent do the home context, the quality of homeschooling, the nature of the teacher student contact or alternative coping strategies may be able to buffer the negative effects of school closures is a research question of outmost relevance but lies beyond the scope of this paper and remains for future research.

With these results, we add one piece to the puzzle needed to conduct a cost-benefit analysis of the pandemic measures. We interpret our results as a call for caution when considering school closures as a measure to stop a viral spread. Our results should also be seen as a call for action to address the needs of adolescents and their families struggling with the negative consequences of school closures (*44*), implying disturbances to their daily routines as well as a disruption in social contacts and interactions.

## MATERIALS AND METHODS

### Data

An overview of all data sources can be found in table S1.

### Data on school closures

In the absence of a comparative and transparent overview, we processed all state-specific corona protection ordinances and compiled a dataset on the state-specific school closure and reopening strategies (*22*). Each federal state enjoyed educational sovereignty and, thus, decided independently about the school closure and particularly the reopening strategy. Decisions were taken for each school track and grade level separately. Thus, the resulting dataset has entries by state, school track (for simplicity, restricted to the main primary and secondary school forms), and grade levels. We assigned the corresponding start and end dates of school closures to each cell defined by a unique combination of state, school track, and grade level. The end date is specified by when a partial reopening took place. The duration of school closure by state and school track specific grade level is determined by the number of weeks between the start and the end dates (see fig. S1 for the resulting variation by state and grade level differentiated by school track). The varying strategies adopted by the 16 German federal states lead to differences in the timing of closure and reopening and, as such, in the duration of school closure. Overall, priority was given to the graduating students (whose grade level differs by state and school track; see fig. S1 for an overview) who started to return already after 4.7 weeks. Overall, the complex German school system, educational sovereignty, and the different strategies of the federal states lead to a substantial and unique variation within states, grade levels, and school tracks ranging from 4.7 to 13 weeks.

### Data on adolescents’ mental health (COPSY and BELLA)

We make use of a unique, German survey on adolescents’ mental health, the so-called BELLA cohort study. BELLA forms part of the nationwide, longitudinal, representative German National Health Interview and Examination Survey for Children and Adolescents, also referred to as KiGGs [see also (*20*, *33*, *45*)]. Specifically, we rely on (i) the last pre-pandemic wave of BELLA, conducted with *n* = 1480 11- to 17-year-old adolescents between August 2015 and November 2017 in the form of computer adaptive tests, and (ii) the COVID-19 online issue of the survey, also referred to as the COPSY [see also (*19*)], conducted with 1040 11- to 17-year-olds between 26 May and 10 June 2020. In addition, we draw upon data from surveys with *n* = 1040 parents of 11- to 17-year-old adolescents, which were also conducted online between 26 May and 10 June 2020. All participants gave informed consent, and the study was approved by the Local Psychological Ethics Committee of the University of Hamburg (LPEK-0151).

To measure well-being and mental health, we rely on internationally accepted, validated, and comparable measures that are in accordance with the guidelines of the International Consortium for Health Outcomes Measurement (*34*, *46*). Specifically, we use the KIDSCREEN-10 Index and the HBSC-SCL to measure adolescents’ well-being and psychosomatic complaints. The KIDSCREEN-10 Index is based on the KIDSCREEN-52 and constitutes a global measure for HRQoL. It is computed using the responses on a five-point Likert scale (from “never” to “always” or from “not at all” to “extremely”) with 10 questions capturing information, e.g., “Have you felt fit and well during the previous week?”. The KIDSCREEN-10 Index is developed according to the item response theory (international *T* values based on RASCH modeling) (*35*, *47*). The second scale, the HBSC-SCL, contains eight questions assessing the frequency of psychosomatic complaints (e.g., headache and nervousness) within the past week on a five-point response scale (from “not at all” to “daily”) (*36*). We further draw upon three clinical scales. The SDQ provides information about emotions, behaviors, and relationships regarding children and adolescents during the previous week. It contains, in total, 20 items divided in four subscales on emotional (e.g., “Many worries, often seems worried”), conduct (e.g., “Often lies or cheats”), hyperactivity (e.g., “Constantly fidgeting or squirming”), and peer problems (e.g., “Often fights with other children or bullies them”), each providing three response options from “not true” to “certainly true” (*37*). We further use the CES-DC to examine depressive symptoms. This scale is generated on the basis of seven items (e.g., “I felt sad”) with frequency during the previous week scored on a scale of 0 (= “not at all”) to 3 (= “a lot”) (*38*, *48*). Last, we rely on the nine-item generalized anxiety subscale of the German SCARED. Here, adolescents are asked to score statements such as “I am nervous” with three response options (from 0 = “not true or hardly ever true” to 2 = “very true or often true”) (*39*, *49*). All scales stem from the youth survey except the SDQ provided by the parental questionnaire.

To identify the effects on parental mental health and FC, we use two scales stemming from the parental questionnaire. First, we rely on the PHQ-8 measuring parental depressive disorder. This scale summarizes eight statements (on a four-response scale from “not at all” to “nearly every day”) regarding personal health (e.g., “Feeling down, depressed, or hopeless,” “Troubles falling asleep or staying asleep, or sleeping too much,” and “Feeling tired or having little energy”) (*40*). Second, we draw upon the parent-reported FC scale assessing family climate with four statements as “In our family, everybody cares about each other’s worries” on a four-response scale from “not true” to ‘exactly true” (*41*).

### Merged dataset (COPSY, pre-pandemic wave of BELLA, and school closure data)

We can merge the self-compiled dataset on school closure (*22*) with the COPSY and BELLA data via adolescents’ state, grade level, and school track. The resulting COPSY sample contains in *n* = 907 11- to 17-year-old adolescents [age mean, 14.2; SD, 1.8; see table S4, representative for German youth (132 observations are dropped because of missing information on the state and/or school track)]. Interviews took place while some of the adolescents were still at home. Because we lack the exact survey date, we take the start date of COPSY, 26 May 2020, to impute a conservative measure of individual duration of school closure for these cases. Using this imputation method, adolescents in our sample experience school closure lasting at least 4.7 and at most 10.1 weeks. Using an alternative imputation method, the end date of COPSY, 10 June 2020, to define the individual duration of school closures, results in a maximum duration of 12.3 weeks. The resulting BELLA sample contains *n* = 1334 11- to 17-year-old adolescents [age mean, 13.8; SD,1.7; see table S5, representative for German youth (155 observations are dropped because of missing information)]. The KIDSCREEN-10 and the CES-DC are standardized to mean of 0 and SD of 1 in BELLA. As the remaining three measures are only elicited in COPSY (but not in BELLA), we standardized them to mean of 0 and SD of 1 in COPSY.

### Crisis helpline call data

We also rely on data from the “Kinder- und Jugendtelefon,” a dedicated phone helpline service for children and adolescents, operated by the nonprofit organization Nummer gegen Kummer e.V. The service is supported by Deutsche Telekom AG, with additional funding provided by the German Federal Ministry for Family Affairs, Senior Citizens, Women and Youth, as well as by the European Union and the Stiftung Deutsche Kinder-, Jugend- und Elterntelefone. The helpline is free of charge; calls are answered from Monday to Saturday between 2 p.m. and 8 p.m. by around 3200 trained volunteer counselors. We have access to all calls entering between January 2019 and December 2020 that developed into deeper conversations and counseling (*50*). The helpline guarantees anonymity to their callers, and it is impossible to identify callers from conversation-level data that we have at hand. However, callers are informed that anonymous call data are collected for reporting and statistical purposes, explicitly in the terms and conditions and implicitly in annual reports and online publications. Further information is available online at www.nummergegenkummer.de.

Information on detailed, nonexclusive conversation topics allows us to track the importance of problems among the vulnerable population of callers. Counselors report the age of callers if stated during the conversation or provide an estimate, allowing us to approximate the most likely grade level for each caller. Together with information on the location of the receiving helpline center, we link the call data with our data on school closure by the federal state and the approximated grade level. Because we do not have information on the school track, we use the average school closure for the respective grade level. We focus on calls by callers of ages 11 to 17 to increase the comparability with the previous analyses based on survey data. The overall sample amounts to *n* = 126,006 calls of 11- to 17-year-old adolescents, of which 51,833 are informative about the reasons why adolescents are calling (the remaining calls relate mainly to unspecified psychosocial and health issues; for more details, see table S6).

### Data on further pandemic measures

We consider the following pandemic measures (*30*). These measures are all measures with meaningful variation until 25 May 2020 across states. We disregard pandemic measures with negligible variation until 25 May 2020 across states, such as travel restrictions, mask or test mandates, work from home recommendations or other workplace restrictions, curfews, and distancing rules.

• Private spaces: The mildest restriction on contacts and gatherings in private spaces is a recommendation to avoid contacts. More stringent versions of this measure impose a maximum number of people gathering in private spaces.

• Public spaces: The mildest restriction on contact and gatherings in public spaces is a recommendation to avoid contacts in public spaces. More stringent versions of this measure impose a maximum number of people gathering in public spaces.

• Indoor events: The mildest level of restriction restricts public indoor events to a maximum of 1000 people. The increasing levels reduce the maximum number of people until the highest level of restriction bans any public indoor event.

• Outdoor events: The mildest level of restriction restricts public outdoor events to a maximum of 5000 people. The increasing levels reduce the maximum number of people until the highest level of restriction bans any public outdoor event.

• Institutions: The mildest level of restriction imposed on educational and cultural institutions involves explicit hygiene rules. The intermediate levels restrict the maximum number of people, allow only outdoor institutions to open, restrict the sale of drinks and food, or ban any institution to open except for museums. The highest level of restriction bans any institution to open.

• Retail and wholesale: The mildest level of restriction imposes hygiene rules. The intermediate levels restrict opening hours or ban large shops to open. The highest level of restriction bans any noncritical retail and wholesale to open.

• Gastronomy: The mildest level of restriction imposes hygiene rules. The intermediate levels restrict opening hours, ban indoor consumption, allow only to-go, and require an appointment, or a combination thereof. The highest level of restriction bans any gastronomy.

• Services and crafts: The mildest level of restriction imposes hygiene rules. The intermediate levels restrict any services with unavoidable customer contact with exceptions for hair saloons or health and care services. The highest level of restriction bans any services and crafts.

• Nightlife: The mildest level of restriction imposes hygiene rules. The intermediate levels ban clubs but not bars to open. The highest level of restriction bans any nightlife venue to open.

• Accommodations: The mildest level of restriction imposes hygiene rules. The intermediate levels ban the accommodation of tourists. The highest level bans any accommodation.

• Indoor sports: The mildest level of restriction bans tournaments with spectators. The intermediate levels restrict the maximum number of people or bans sports with physical contact. The highest level bans any indoor sports.

• Outdoor sports: The mildest level of restriction restricts the maximum number of people for outdoor sports. The intermediate levels ban outdoor sports with physical contact. The highest level bans any use of outdoor sport facilities.

Each pandemic measure has different levels. The definition of measures and their levels are taken from (*30*). The stringency of each measure is calculated as follows: For each day, from 1 March to 25 May, all levels are summed up for each state and measured separately. Then, the population-weighted median level restriction is determined, and states are split accordingly. Similarly, we calculate how many people per capita have been infected until 25 May. Then, we calculate the population-weighted median. We do the very same for the number of deaths per capita caused by COVID-19. On both measures, the population-weighted median split assigns the same group of states in the high and low groups.

### Short-run impact of school closures and their contribution to the overall deterioration of mental health

We use a linear regression model with two levels of fixed effects (*28*, *29*, *51*), to identify the effect of school closures on adolescents’ mental health. The variable of interest, the individual duration of school closures, is perfectly determined by the combination of the individual state and the school track–specific grade level. This two-way fixed-effects method accounts for two levels of fixed effects, a set of state- and school track–specific grade level fixed effects, and thus absorbs any level differences between states and school track–specific grade levels in adolescents’ mental health. Identification is thus based on the remaining variation in the duration of school closures within the states (across school track–specific grade levels) and within school track–specific grade levels (across states), which is arguably exogenous and thus serves as a framework for a quasi-experiment. The underlying identifying assumption is that there are no systematic, confounding factors driving the deviation in adolescents’ mental health from the mental health predicted for any adolescents residing in state *s* and attending the school track–specific grade level cx, other than the duration of school closure. This implies that neither the pandemic severity (e.g., infection rates, hospitalization, and death rates) nor further pandemic measures (e.g., contact restrictions and home office) vary within states across school track–specific grade levels (*30*). This assumption is plausible as (i) case rates among adolescents were negligible (at least in the first wave of the pandemic) and case rates and deaths of parents or grandparents should be comparable across the age ranges of the children in our sample; and (ii) there are no further pandemic measures targeting explicitly specific age groups, grade levels, or schools (*30*).

More formally, we model youth mental health using the following equation

$$y_{iscx}=\alpha +\beta d_{scx}+\gamma_{s}+\gamma_{cx}+X_{i}^{\prime }\delta +\varepsilon_{iscx}$$ (1)

where *y_iscx_* constitutes the dependent variable, comprising the different measures of the mental health of individual *i* that lives in state *s* and attends school track *x* in grade *c*. We standardize all outcome variables to a mean of 0 and SD of 1, facilitating the comparison across different mental health dimensions and the interpretation of the effect size. The independent variable *d_scx_* denotes weeks of school closure applying to all individuals residing in state *s*, attending grade level *c* in school track *x*. We include a constant α as well as state (γ*_s_*) and school track–specific grade level (γ*_cx_*) fixed effects. We further control for adolescents’ age (in years) and sex (using a dummy = 1 if female) summarized by the matrix *X_i_*. ɛ*_iscx_* represents an idiosyncratic error term. All estimates shown in Fig. 2A and tables S7A and S8 result from estimation equation (see Eq. 1) using ordinary least square and clustering the SEs at the state*school track*grade level and thus on treatment level (*52*).

The following example of two neighboring states helps illustrating the idea underlying this empirical approach: Bavaria gave priority to entry grade levels with the higher grade levels following only subsequently. Thus, in Bavaria, a fifth grader (the entry grade level in secondary school) returned to school by 18 May 2020, while a sixth grader returned only by 15 June 2020 (4 weeks later). In contrast, the neighboring state Baden-Württemberg reopened schools for all lower grade levels in secondary schools (fifth, sixth, seventh, and eighth graders) “en bloc” on 15 June 2020. Comparing fifth and sixth graders in Bavaria nets out any state-specific effects of the pandemic and its related measures and thus leaves us with the mental health differences not only because of the additional 4 weeks school closures but also possibly because of age differences in mental health and in the way how differently aged adolescents dealt with the pandemic and its measures. Comparing fifth and sixth graders in Baden-Württemberg, in turn, allows determining the age differences in mental health during the COVID-19 pandemic, again, net of any state-specific effects of the pandemic or its related measures. The double comparison isolates the effect of the four additional weeks school closure net of state-, grade-, and school track–specific differences in adolescents’ mental health during the COVID-19 pandemic.

When drawing on both datasets, the COPSY data and pre-pandemic wave of BELLA, we exploit the different time periods and rely on the following two specifications

$$y_{iscxt}=\alpha +\beta_{2}c_{t}+X_{it}^{\prime }\delta +\varepsilon_{iscxt}$$ (2)

$$y_{iscxt}=\alpha +\beta_{1}d_{scx}+\beta_{2}c_{t}+\beta_{3}c_{t}\times d_{scx}+X_{it}^{\prime }\delta +\varepsilon_{iscxt}$$ (3)

where *y_iscxt_* constitutes the dependent variable, comprising the different measures of the mental health of individual *i* in state *s* in grade *c* that attends school track *x* at time *t* (which can take two values: pre-pandemic or during the pandemic). The independent variable *d_scx_* denotes the weeks of school closure mandated during the pandemic in the state where child *i* resides and for the grade level and school track that child *i* attends. Note that this only reflects the state that child *i* lives in as well as the school track and the grade level that he/she attends. Whether a child suffered from the mandated weeks of school closure depends on whether the child is observed before or after the outbreak of the pandemic (and thus whether the observation belongs to BELLA or COPSY data). *c_t_* is a dummy that is 1 for any observation belonging to COPSY and, thus, when schools were closed and is 0 for any observation belonging to BELLA. We further control for adolescents’ age (in years) and sex (using a dummy = 1 if female) summarized by the matrix *X_it_* and include a constant α. ɛ*_iscxt_* represents an idiosyncratic error term.

Using this additional estimation model, we learn about the following parameters: The effect of one additional week of school closure is given by β_3_. The overall COVID-19 effect net of school closures is given by β_2_. β_1_ captures preexisting level differences in adolescents’ mental health and to which extent they may be correlated with the mandated weeks of school closures. As such, the estimated β_1_ provides us with some insights whether our identifying assumption—the weeks of school closures is independent of children’s mental health and as such ability to cope mentally with the school closures—applies. The results of this specification are shown in table S7C, and β_2_ is presented as red bars in Fig. 2B. The results for the overall deterioration in the various mental health measures over the pandemic (resulting from estimating Eq. 2 but dropping *d_i_* and *c_t_ × d_i_*) are shown in table S7B and as blue bars in Fig. 2B.

### Sensitivity checks

To check the sensitivity of our baseline results, we run a series of alternative specifications presented in table S8 (B to H) We first list the baseline results resulting from estimating Eq. 1 (table S8A). The remaining panels show the estimates resulting of the various robustness checks regarding our baseline specification. First, we use a more parsimonious approach and exclude all individual control variables contained in the vector (table S8B). In table S8C, we aim at absorbing any level differences in adolescents’ mental health across school tracks within states and thus control for a fully interacted set of state and school track fixed effects (instead of a set of state fixed effects only). In table S8D, we include the second-order polynomial of weeks of school closure as a further covariate in Eq. 1 to allow for any nonlinear effects. In table S8 (E and F), we reconsider the duration of school closures. In table S8E, we use the survey end date (instead of the survey start date) to impute the duration of school closure for all adolescents that had not returned to school before 26 May 2020 (the start of COPSY). In table S8F, we adjust the duration of school closure for any school holidays taking place during the lockdown. Here, we subtract the weeks of vacations from the duration of school closure to calculate the weeks of school closure. In table S8G, we use parental reports on whether their adolescent child had returned to school or still lingered in homeschooling instead of the mandated weeks of closures based on individual state of residence, grade level, and school track. For this sensitivity check, we rely on a dummy variable taking the value of 1 if teaching takes place mainly or exclusively at home. Following the recommendation to use both self-reported and externally evaluated answers to mental health scales (*53*, *54*), we reestimate Eq. 1 using parental reports on adolescents’ mental health as the dependent variable (see table S8H). We can do so only for the KIDSCREEN-10 index and HBSC-SCL scale, as the parental questionnaire does not contain the further screening devices for mental health problems. For comparability, we restrict the sample to parents reporting on their adolescent children only (age interval of 11 to 17). In table S8I, we assess whether our results are driven by seasonality in pre-pandemic data when estimating Eq. *3*. For that, we include dummies for the specific quarter in which the interviews were conducted. In table S8J, we address the concern of possible time trends in HRQoL or CES-DC by including a linear time trend (measured in years).

### Sensitivity checks including pandemic severity and stringency of pandemic measures

To estimate the effect of state-level pandemic severity and stringency of pandemic measures, we need to deviate from our baseline equation (see Eq. 1) and sacrifice controlling for the set of state dummies (as these absorb anything that is constant at the state level). We therefore estimate the correlated random effects model in Eq. 4, following the Chamberlain-Mundlak approach. This approach keeps dummy variables for school track–specific grade level (Γ*_cx_*) and control variables for adolescents’ age (in years) and sex (using a dummy = 1 if female) summarized by the matrix *X_i_*. The Chamberlain-Mundlak approach adds the average state-level weeks of school closure *d*^¯^*scx*, the average grade-track dummies by state Γ^¯^_*scx*_, and the average state-level age and gender *X*^¯^_*scx*_. The advantage of this approach is that it allows us to control for additional state-level variables. Note that the underlying idea of this approach resembles the idea of the two-way fixed-effects approach shown in Eq. 1 as both rely on netting out state-level averages of all independent variables and are identified by deviations from the state-level mean. As such, the resulting estimates for the effect of weeks of school closure β are comparable across the two approaches (see tables S7A and S9A).

$$y_{iscx}=\alpha +\beta \;d_{scx}+\zeta {\bar{d}}_{scx}+\lambda m_{s}+{\text{Γ}}_{cx}\eta +{\bar{\text{Γ}}}_{scx}^{\prime }\theta +X_{i}^{\prime }\;\delta +{\bar{X}}_{scx}^{\prime }\;\iota +\varepsilon_{iscx}$$ (4)

We then go on and add consecutively dummies indicating the state-level stringency in a series of pandemic measures (please refer to table S3 for a classification of the states in the various pandemic measures). To do so, we include a dummy variable *m_s_* which is equal to 1 when the respective state has high restrictions (at and above the median level) on a certain pandemic measure and 0 otherwise. The estimates for λ can be interpreted as the effect the respective pandemic measure has on youth mental health above and beyond the effect of prolonged school closures.

### Subgroup analysis

For the subgroup analysis in Fig. 3 and table S10, we adapt the baseline model of Eq. 1 and add interaction terms between the weeks of school closures and dummy variables for the respective subgroups. Note that the respective main effects are already included in *X_i_*. To examine the effect of school closures by adolescents’ age (shown in Fig. 3A and table S10A), we add the interaction terms between weeks of school closure *d_scx_* and a full set of age dummies (μ*_ij_*, which is equal to 1 if the individual *i* is aged *j* and 0 otherwise). Note that this model (see Eq. 5) allows us to measure the effect of weeks of school closure on mental health for each age group *j* separately.

$$y_{iscx}=\alpha +\sum_{j=11}^{17}\beta_{j}\mu_{ij}\;d_{scx}+\gamma_{s}+\gamma_{cx}+X_{i}^{\prime }\;\delta +\varepsilon_{iscx}$$ (5)

In Fig. 3B and table S10B, we include the interaction of weeks of school closure and the female dummy μ*_if_* as well as the interaction of weeks of school closure and the male dummy μ*_im_*. This allows us to identify the effect of weeks of school closure on mental health for boys (β*_m_*) and girls (β*_f_*) separately

$$y_{iscx}=\alpha +\beta_{m}\mu_{im}d_{scx}+\beta_{f}\mu_{if}d_{scx}+\gamma_{s}+\gamma_{cx}+X_{i}^{\prime }\;\delta +\varepsilon_{iscx}$$ (6)

Last, we show the effect by living space per school-aged child (see Fig. 3C and table S10C). For this purpose, we divide the size of the apartment or house in square meters by the number of children in the household. Then, we do a median split and create two dummy variables: μ*_ia_* that is equal to 1 if a child has a living space above the median and 0 otherwise; and μ*_ib_* that is equal to 1 if a child has a living space below the median and 0 otherwise. We then estimate an augmented model where we add these two dummy variables indicating living space per-school age child as well as their interactions with the mandated weeks of school closure to the baseline equation (see Eq. 7). This allows us to identify the effect of weeks of school closure on mental health for children having a lot of space (β*_a_*) and children having less space (β*_b_*) separately

$$y_{iscx}=\alpha +\beta_{a}\;\mu_{ia}\;d_{scx}+\beta_{b}\;\mu_{ib}\;d_{scx}+\gamma_{b}+\gamma_{s}+\gamma_{cx}+X_{i}^{\prime }\delta +\varepsilon_{iscx}$$ (7)

### Crisis helpline call data presentation

The development of helpline calls is presented in Fig. 4 (A and B). For that, we take all calls in 2019 and 2020 and estimate the following model

$$y_{t}=\alpha +\sum_{m=1}^{11}\gamma_{m}+\gamma_{\mathrm{sat}}+\gamma_{\mathrm{sun}}+\varepsilon_{t}$$ (8)

where *y_t_* corresponds to the total length of all calls for day *t* and a given topic and group. γ*_m_* is a set of month dummies, while γ_sat_ and γ_sun_ are dummies accounting for calls coming in over the weekend. In Fig. 4 (A and B), we display the deviation from the predicted trend using the 2019 data using the residuals *e_t_* = *y_t_* − ${\hat{y}}_{t}$ and standardizing them to have mean of 0 and SD of 1 in 2019. We further calculate a 28-day moving average from *e_t_* to *e*_*t−*27_. The moving SD and the corresponding confidence interval are based on the same 28 days.

### Crisis helpline call data analysis

To account for the autocorrelation of the error term in helpline calls, we estimate the following model

$$y_{t}=\alpha +\sum_{i=1}^{22}\beta_{i}\;\mu_{i}+\sum_{m=1}^{11}\gamma_{m}+\gamma_{\mathrm{sat}}+\gamma_{\mathrm{sun}}+\sum_{j=1}^{7}\rho_{j}y_{t-j}+\theta \varepsilon_{t-1}+\varepsilon_{t}$$ (9)

where *y_t_* is the total duration of calls at day *t* for a certain group and topic. μ*_i_* is a set of dummies indicating 14 day windows where μ_1_ starts on Monday, 30 December 2019, and ends on Sunday, 12 January 2020, and μ_22_ starts on Monday, 19 October 2020, and ends on Sunday, 1 November 2020. The dummies γ*_m_*, γ_sat_, and γ_sun_ are the same as in Eq. 8. $\sum_{j=1}^{7}\rho_{j}$*y_t−j_* is a 7-day moving average, and θɛ_*t−*1_ allows for an autoregressive process of order 1. All β*_i_* estimates are presented in table S12, and most β*_i_* estimates are shown in fig. S3. We abstain from reporting longer periods, as the infection rates started increasing rapidly in the autumn, resulting in a high number of local school closures and, ultimately, in the second phase of nationwide school closures, rendering an analysis of the mental health effects caused by the initial school closures going beyond 2020.

## Supplementary Materials

### This PDF file includes:

Figs. S1 to S3 Tables S1 to S12 References
