# Supplementary material for: The youth mental health crisis: Quasi-experimental evidence on the role of school closures
Source: Sci Adv. 2023 Aug 18;9(33):eadh4030. doi: 10.1126/sciadv.adh4030 (PMC10438447; doi:10.1126/sciadv.adh4030)
Supplement: Supplementary file 1 — Figs. S1 to S3 Tables S1 to S12 References [file sciadv.adh4030_sm.pdf]

Science Advances
AAAS

Supplementary Materials for

The youth mental health crisis: Quasi-experimental evidence on the role of school closures

Christina Felfe *et al.*

Corresponding author: Christina Felfe, christina.felfe@uni-konstanz.de;
Ulrike Ravens-Sieberer, ravens-sieberer@uke.de

Sci. Adv. **9**, eadh4030 (2023)
DOI: 10.1126/sciadv.adh4030

This PDF file includes:

Figs. S1 to S3
Tables S1 to S12
References

Figures

Figure S1: Variation in school closure and re-opening strategies by tracks

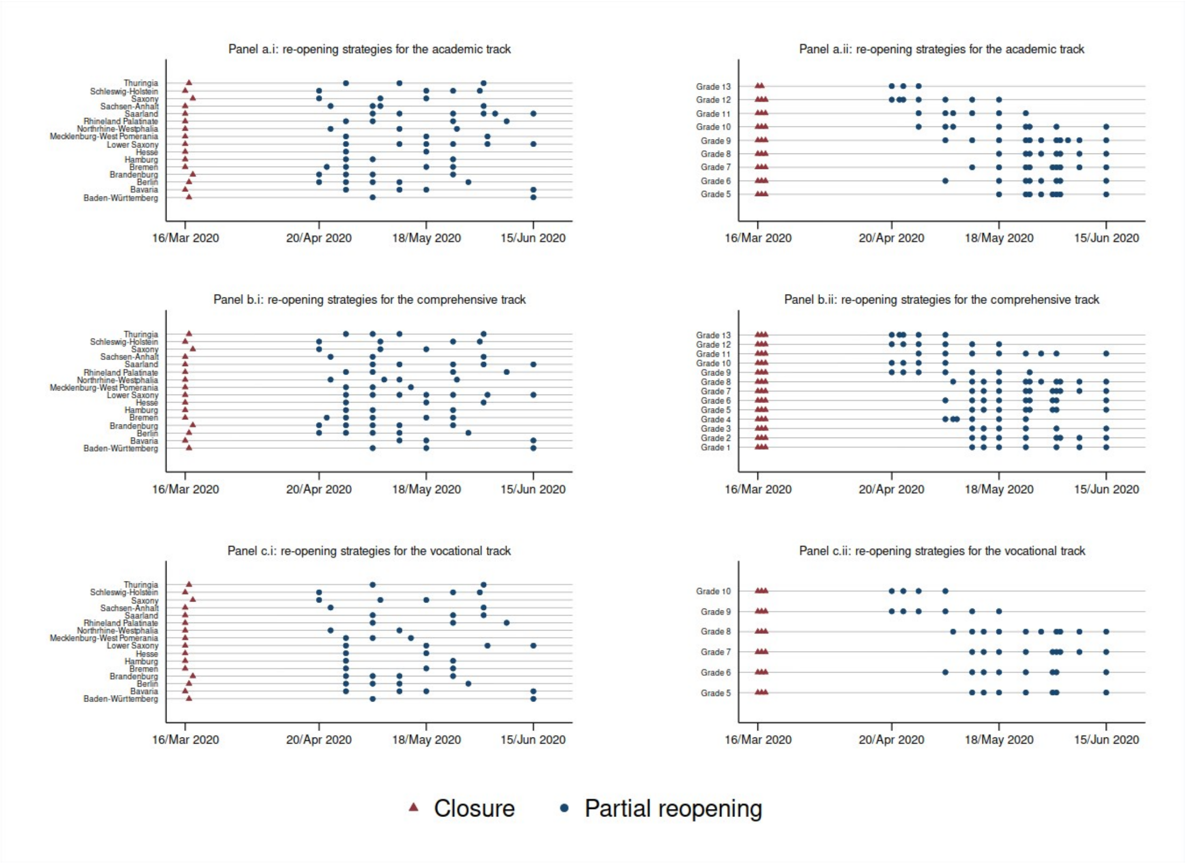

Figure S1: Variation in school closure and re-opening strategies by (i) states and (ii) grade levels, separately for the academic track (Panel a), the comprehensive track (subsuming various types of primary and secondary schools) (Panel b), and the vocational track (Panel c). Data are compiled by screening the state-specific Corona protection ordinances (*22*). Each blue dot represents the date when the respective federal government (partially) re-opened schools for selected grade levels (Panel i) and the resulting variation in the re-opening dates by grade levels across the federal states (Panel ii).

Figure S2: Pre-pandemic levels of mental health by state and grade

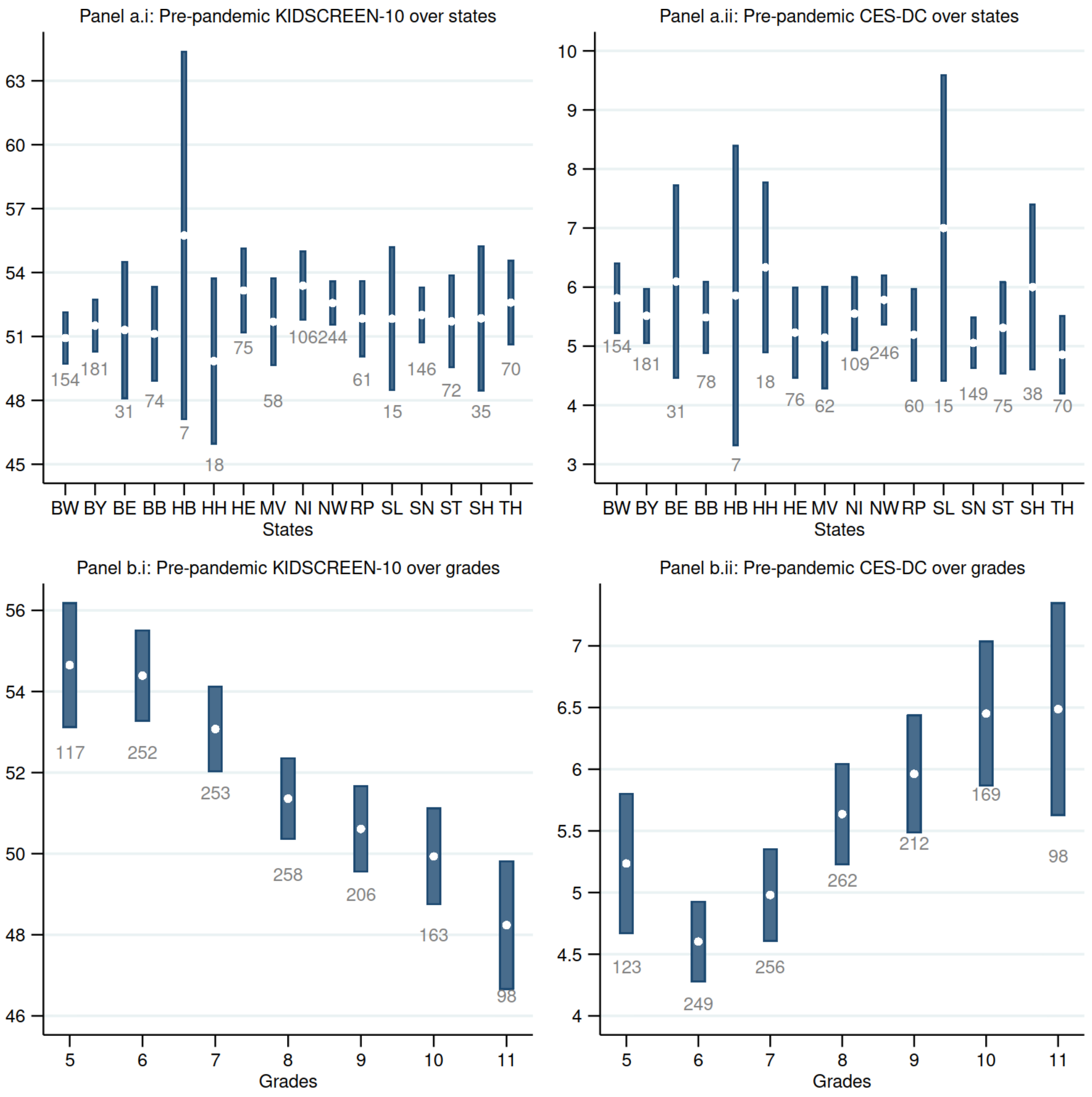

Figure S2: Shows the means (including the 95% confidence interval) of pre-pandemic mental health in 2017 over states and grades. Baden-Württemberg (BW), Bavaria (BY), Berlin (BE), Brandenburg (BB), Bremen (HB), Hamburg (HH), Hesse (HE), Mecklenburg-West Pomerania (MV), Lower Saxony (NI), Northrhine-Westphalia (NW), Rhineland Palatinate (RP), Saarland (SL), Saxony (SN), Saxony-Anhalt (ST), Schleswig-Holstein (SH) and Thuringia (TH).

Figure S3: ARMA estimation of family problems by school closure in 2020

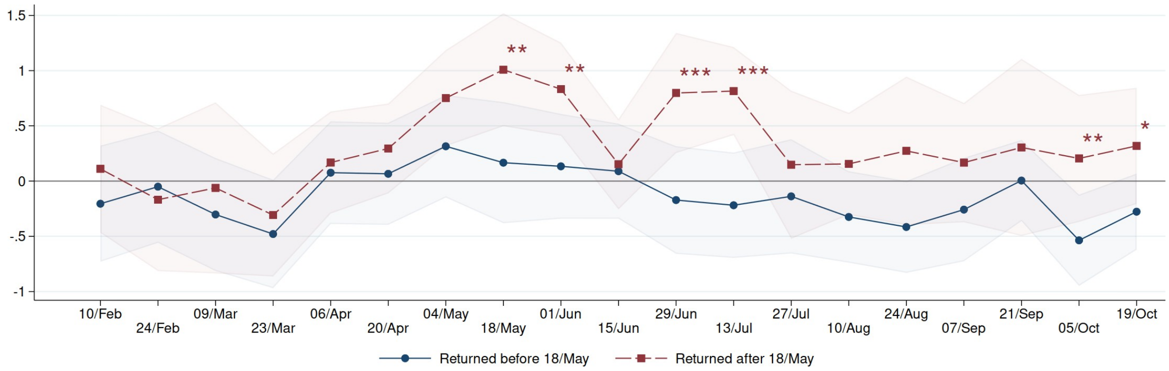

Figure S3: Shows the coefficient estimates of one regression for each group of daily call volume (number of calls times their duration standardized to have mean 0 and sd of 1 for 2019) on a set two-weeks time dummies with an autoregressive moving-average process of order 1 and 7, respectively, and a set of month dummies and weekend dummies. The stars indicate the significance level of a test on the equality of the two coefficients. $^{*}\ p < 0.1$, $^{**}\ p < 0.05$, $^{***}\ p < 0.01$ See equation (9) and Table S12 in Materials and Methods for details.

Tables

Table S1: Data sources

	School closure data[1]	COPSY[2]	BELLA[3]	Crisis helpline[4]
Panel a: purpose				
	Treatment	Main outcomes	Pre-pandemic outcomes	Mechanisms
Panel b: data collection				
Start	Mar 16, 2020	May 26, 2020	Aug 2015	Jan 1, 2019
End	Jun 15, 2020	Jun 10, 2020	Nov 2017	Dec 31, 2020
Type	archival	online interviews	in-person interviews	administrative
Panel c: population				
Used observations	422 treatments	907 adolescents<br>907 parents	1,334 adolescents	126,006 calls<br>of adolescents
Sample	population	representative	representative	population
Panel d: variables				
Unit of observation	State × Grade × School track	Individual	Individual	Individual calls
Variables	Date school closure	KIDSCREEN-10	KIDSCREEN-10	Topic
	Date school opening	HBSC-SCL		Call duration
	Weeks of school closure	SDQ		Call date
		CES-DC	CES-DC	
		SCARED		
		PHQ-8		
		FC		
		Age	Age	Age
		Gender	Gender	Gender
	Grade	Grade	Grade	
	School track	School track	School track	
	State	State	State	State

Notes: The data originates from the following sources: [1] (*22*), [2] (*19*), [3] (*20*), and [4] (*50*)

Table S2: Comparison of pandemic severity and stringency of pandemic measures

Panel a: comparison by state

State	Total cases per million	Total deaths per million
Baden-Württemberg	3121	168
Bavaria	3548	199
Berlin	1818	57
Brandenburg	1278	66
Bremen	1922	70
Hamburg	2730	145
Hesse	1562	83
Mecklenburg-West Pomerania	472	12
Lower Saxony	1454	79
Northrhine-Westphalia	2080	94
Rhineland Palatinate	1609	58
Saarland	2678	172
Saxony	1299	56
Saxony-Anhalt	770	28
Schleswig-Holstein	1056	52
Thuringia	1353	87

Panel b: international comparison

Country/Region	Total cases per million	Total deaths per million	Stringency index
Austria	1477	72	39
Denmark	1931	97	38
France	2257	438	48
**Germany**	**2145**	**109**	**40**
Italy	3893	555	56
Netherlands	2561	330	40
Poland	535	25	42
Spain	5101	605	45
Switzerland	3514	197	37
United Kingdom	3615	745	40
Europe	2439	247	-
United States	4908	294	39

Panel a: Column 1-2 contain the cumulated numbers up to May 25, 2020 using the following variables: total confirmed cases of COVID-19 per million people (*57*) and the total COVID-19 deaths per million people (*58*). **Panel b:** Column 1-2 contain the cumulated numbers up to May 25, 2020 using the following variables: The total confirmed cases of COVID-19 per million people (*56*), total deaths attributed to COVID-19 per million people (*56*). Column 3 contains the average Oxford stringency index (This is a daily calculated index taking values between 0 and 100, where a higher score indicates stricter government policies, such as: school closing, workplace closing, cancel public events, restrictions on gathering size, close public transport, stay-at-home requirements, restrictions on internal movement, restrictions on international travel, and public information campaign (*56, 55*)).

Table S3: State-level comparison of pandemic severity and stringency of pandemic measures up to May 25, 2020

	BW	BY	BE	BB	HB	HH	HE	MV	NI	NW	RP	SL	SN	ST	SH	TH
Pandemic severity	high	high				high				high		high				
Restrictions on																
Private spaces	high	high	high	high							high	high	high	high	high	
Public spaces	high			high		high	high			high		high		high		
Indoor events		high				high				high	high		high	high		
Outdoor events		high				high	high			high	high			high		
Institutions	high	high		high		high	high				high	high		high	high	
Retail, wholesale			high	high		high	high	high		high		high	high	high	high	
Gastronomy			high			high	high		high	high	high	high	high	high	high	
Services, crafts	high			high		high	high		high		high	high		high	high	high
Nightlife	high			high		high				high		high	high	high	high	
Accommodations	high		high	high		high	high	high		high		high			high	
Indoor sports			high	high		high	high			high		high	high	high	high	
Outdoor sports	high			high		high	high			high		high		high	high	

Notes: We categorize each state according to the pandemic severity (measured by cases rates and deaths per capita) and the stringency of the overall pandemic measures in place: ”high” implies a severity or stringency of the pandemic measures at or above the median level of the respective measure. To calculate the median value, we weight all observations by the state-level population. Detailed descriptions on the pandemic measures are in Materials and Methods. Additional measures with no variation across states were as follows: travel restrictions, mask or test mandates, work from home recommendations or other workplace restrictions, curfews, distancing rules. State abbreviations are as follows: Baden-Württemberg (BW), Bavaria (BY), Berlin (BE), Brandenburg (BB), Bremen (HB), Hamburg (HH), Hesse (HE), Mecklenburg-West Pomerania (MV), Lower Saxony (NI), Northrhine-Westphalia (NW), Rhineland Palatinate (RP), Saarland (SL), Saxony (SN), Saxony-Anhalt (ST), Schleswig-Holstein (SH) and Thuringia (TH)

Table S4: Descriptive statistics of estimation sample with COPSY data

	N	Mean	SD	Min	Max
Panel a: outcome variables					
HRQoL					
KIDSCREEN-10	907	45.35	8.24	22	84
Screening devices for adolescents' mental health problems					
HBSC-SCL	907	-35.99	4.49	-40	-12
SDQ	907	8.85	5.53	0	32
CES-DC	907	6.28	3.62	2	22
SCARED	907	14.54	4.27	9	27
Screening devices for parents' mental health problems					
PHQ-8	907	5.03	4.81	0	24
Family climate					
FC	907	12.84	2.10	4	16
Panel b: treatment variable					
Weeks of school closure	907	8.66	1.63	4.7	10.1
Panel c: sociodemographic background characteristics					
Age (in years)	907	14.16	1.79	11	17
Female (=1, =0 otherwise)	907	0.52	0.50	0	1
Number of children in household	907	1.48	0.68	1	5
Living space (in sqm)	907	126.57	54.35	38	450
School tracks					
Vocational (=1, =0 otherwise)	907	0.36	0.48	0	1
Comprehensive (=1, =0 otherwise)	907	0.23	0.42	0	1
Academic (=1, =0 otherwise)	907	0.41	0.49	0	1

Notes: Number of observations, mean, standard deviation, minimum, and maximum of the main sociodemographic factors and outcome variables of the unweighted final dataset are presented. Weights are developed based on the current German Microcensus (2018). The sample consists of n=907 11-17-year-old adolescents participating in COPSY (May 26 - June 10, 2020).

Table S5: Descriptive statistics of estimation sample with BELLA data

	N	Mean	SD	Min	Max
Panel a: outcome variables					
HRQoL					
KIDSCREEN-10	1334	52.02	8.57	25	84
Screening devices for adolescents' mental health problems					
CES-DC	1334	5.50	3.41	2	23
Panel b: sociodemographic background characteristics					
Age (in years)	1334	14.02	1.80	11	17
Female (=1, =0 otherwise)	1334	0.53	0.50	0	1
School tracks					
Vocational (=1, =0 otherwise)	1334	0.28	0.45	0	1
Comprehensive (=1, =0 otherwise)	1334	0.16	0.37	0	1
Academic (=1, =0 otherwise)	1334	0.56	0.50	0	1

Notes: Number of observations, mean, standard deviation, minimum, and maximum of the main sociodemographic factors and outcome variables of the unweighted final dataset are presented. Weights are developed based on the current German Microcensus (2018). The sample consists of n=1'334 11-17-year-old adolescents participating in the pre-pandemic wave of BELLA (August 2015 - November 2017).

Table S6: Descriptive statistics of crisis helpline call data

Main focus of call	No. of calls	Call duration		Age		Male
		Mean	SD	Mean	SD	Share
Parents and siblings	22,470	15.8	13.4	14.3	1.8	49.1%
School and teachers	8,824	14.7	13.0	14.2	1.9	51.9%
Friends and peers	26,455	11.3	10.1	13.6	1.7	57.3%
Σ	51,833	13.0	11.6	13.9	1.8	53.7%
All calls	126,006	12.3	11.2	14.3	1.8	56.9%

Notes: This table shows the number of calls in 2019 and 2020. Each call can have multiple focuses which is why some calls get recorded in multiple categories. Σ gives the number of calls that are recorded in at least one of the three categories above. Call duration is given in minutes and the duration of each call is recorded in increments of 5 minutes.

Table S7: Baseline results for adolescents' HRQoL and mental health

	KS-10	HBSC-SCL	SDQ	CES-DC	SCARED
Panel a: average effect (based on equation (1))					
Weeks of school closure	-0.107***	0.072**	0.089**	0.078**	0.030
	(0.030)	(0.034)	(0.038)	(0.037)	(0.034)
Panel b: overall Covid effect (based on equation (2))					
Covid	-0.837***			0.243***	
	(0.048)			(0.050)	
Panel c: average effect when accounting for pre-Covid levels (based on equation (3))					
Weeks of school closure	-0.001			-0.016	
	(0.023)			(0.024)	
Weeks of school closure × Covid	-0.086***			0.076**	
	(0.028)			(0.031)	
Covid	-0.085			-0.430	
	(0.252)			(0.286)	

Notes: Each column in a panel is a separate regression. KIDSCREEN-10 and CES-DC have mean 0 and sd 1 in BELLA. All remaining variables have mean 0 and sd 1 in COPSY since those are not available in BELLA. Panel a: Regressions use only COPSY with a total of 907 observations. Panel b and c: Regressions use COPSY and BELLA data with a total of 2'232 observations. Regressions include fixed effects for states and grade level school track. All regressions include control variables for age and sex and standard errors are clustered at the state×school track×grade level. $^{*}p < 0.1$, $^{**}p < 0.05$, $^{***}p < 0.01$ For more details see the paragraph on analysis of the impact of school closures.

Table S8: Sensitivity Analysis regarding adolescents' HRQoL and mental health

	KS-10	HBSC-SCL	SDQ	CES-DC	SCARED
Panel a: average effect					
Weeks of school closure	-0.107***	0.072**	0.089**	0.078**	0.030
	(0.030)	(0.034)	(0.038)	(0.037)	(0.034)
Panel b: no control variables (i.e. age and gender)					
Weeks of school closure	-0.105***	0.066*	0.091**	0.073**	0.021
	(0.031)	(0.034)	(0.038)	(0.036)	(0.034)
Panel c: school-track×state and school-track×grade fixed effects					
Weeks of school closure	-0.100***	0.062**	0.102**	0.093**	0.013
	(0.036)	(0.030)	(0.047)	(0.044)	(0.040)
Panel d: second order polynomial of school closure duration					
Weeks of school closure	0.210	-0.354	-0.641*	-0.174	-0.040
	(0.236)	(0.298)	(0.356)	(0.303)	(0.248)
(Weeks of school closure)²	-0.019	0.026	0.044**	0.015	0.004
	(0.015)	(0.018)	(0.021)	(0.018)	(0.015)
Panel e: survey end as cutoff					
Weeks of school closure	-0.083***	0.049*	0.090***	0.070**	0.014
	(0.027)	(0.025)	(0.031)	(0.032)	(0.026)
Panel f: school closure duration without holidays					
Weeks of school closure	-0.083***	0.049*	0.082**	0.063*	0.008
	(0.029)	(0.027)	(0.034)	(0.033)	(0.029)
Panel g: home-schooling					
Currently no school	-0.101	0.157	0.045	0.086	0.165*
	(0.098)	(0.099)	(0.105)	(0.119)	(0.094)
Panel h: parent questionnaire					
Weeks of school closure	-0.119***	0.068*			
	(0.036)	(0.037)			

Continued on next page

Table S8 (contd.): Sensitivity Analysis regarding adolescents' HRQoL and mental health

	KS-10	HBSC-SCL	SDQ	CES-DC	SCARED
Panel i: including dummies for the quarter of interview					
Weeks of school closure	-0.000			-0.015	
	(0.023)			(0.024)	
Weeks of school closure × Covid	-0.086***			0.076**	
	(0.029)			(0.031)	
Covid	-0.064			-0.351	
	(0.256)			(0.280)	
Panel j: including a linear time trend					
Weeks of school closure	-0.000			-0.016	
	(0.023)			(0.024)	
Weeks of school closure × Covid	-0.086***			0.077**	
	(0.028)			(0.031)	
Covid	-0.016			-0.423	
	(0.315)			(0.362)	
Linear time trend	-0.018			-0.003	
	(0.051)			(0.051)	

Notes: Each column in a panel is a separate regression. All outcome variables have mean 0 and sd 1. All regressions include control variables for age and sex. In addition, regressions in Panel a include fixed effects for states and school track-specific grades and in Panel i regressions include fixed effects that account for the specific quarter in which the interviews were conducted. The number of observations is 907 except for Panel c for which it is 903 and for Panel i and j for which it is 2'240. Standard errors are clustered at the state×school track×grade level. * $p < 0.1$, ** $p < 0.05$, *** $p < 0.01$ For more details, please refer to Materials and Methods, sensitivity analysis.

Table S9: Results considering explicitly pandemic severity and stringency of pandemic measures

	KS-10	HBSC-SCL	SDQ	CES-DC	SCARED
Panel a					
Weeks of school closure	-0.101***	0.070**	0.090**	0.077**	0.025
	(0.031)	(0.035)	(0.037)	(0.037)	(0.036)
Panel b					
Weeks of school closure	-0.100***	0.068**	0.093**	0.078**	0.026
	(0.031)	(0.034)	(0.037)	(0.037)	(0.036)
Pandemic severity	-0.039	0.115	-0.101	-0.047	-0.070
	(0.116)	(0.152)	(0.131)	(0.151)	(0.104)
Panel c					
Weeks of school closure	-0.101***	0.068**	0.089**	0.080**	0.029
	(0.031)	(0.033)	(0.037)	(0.037)	(0.036)
Private spaces	0.010	0.051	0.018	-0.054	-0.091
	(0.085)	(0.107)	(0.098)	(0.107)	(0.085)
Panel d					
Weeks of school closure	-0.101***	0.070**	0.090**	0.077**	0.025
	(0.031)	(0.035)	(0.037)	(0.037)	(0.036)
Public spaces	0.020	0.044	-0.101	-0.025	-0.031
	(0.075)	(0.091)	(0.082)	(0.094)	(0.071)
Panel e					
Weeks of school closure	-0.099***	0.068**	0.093**	0.079**	0.029
	(0.031)	(0.034)	(0.037)	(0.037)	(0.036)
Indoor events	0.071	-0.056	0.077	0.070	0.131
	(0.090)	(0.113)	(0.100)	(0.119)	(0.093)
Panel f					
Weeks of school closure	-0.099***	0.068**	0.093**	0.080**	0.030
	(0.031)	(0.034)	(0.037)	(0.037)	(0.035)
Outdoor events	0.057	-0.058	0.079	0.086	0.177**
	(0.081)	(0.100)	(0.089)	(0.107)	(0.088)

Continued on next page

Table S9 (contd.): Results considering explicitly pandemic severity and stringency of pandemic measures

	KS-10	HBSC-SCL	SDQ	CES-DC	SCARED
Panel g					
Weeks of school closure	-0.104***	0.069**	0.087**	0.076**	0.022
	(0.031)	(0.034)	(0.037)	(0.037)	(0.036)
Institutions	0.064	0.030	0.066	0.019	0.043
	(0.069)	(0.086)	(0.085)	(0.085)	(0.074)
Panel h					
Weeks of school closure	-0.100***	0.069**	0.090**	0.078**	0.027
	(0.031)	(0.034)	(0.037)	(0.037)	(0.036)
Retail, wholesale	0.024	-0.046	-0.007	0.055	0.124
	(0.100)	(0.119)	(0.111)	(0.126)	(0.102)
Panel i					
Weeks of school closure	-0.101***	0.070**	0.093**	0.077**	0.026
	(0.031)	(0.035)	(0.037)	(0.037)	(0.036)
Gastronomy	-0.015	-0.003	0.101	-0.013	0.038
	(0.083)	(0.095)	(0.095)	(0.122)	(0.094)
Panel j					
Weeks of school closure	-0.102***	0.066*	0.087**	0.080**	0.029
	(0.031)	(0.034)	(0.038)	(0.037)	(0.035)
Services, crafts	0.016	0.086	0.064	-0.058	-0.102
	(0.087)	(0.112)	(0.096)	(0.112)	(0.098)
Panel k					
Weeks of school closure	-0.101***	0.069**	0.092**	0.078**	0.026
	(0.031)	(0.035)	(0.037)	(0.037)	(0.036)
Nightlife	0.017	0.051	-0.102	-0.040	-0.071
	(0.072)	(0.088)	(0.079)	(0.090)	(0.072)

Continued on next page

Table S9 (contd.): Results considering explicitly pandemic severity and stringency of pandemic measures

	KS-10	HBSC-SCL	SDQ	CES-DC	SCARED
Panel l					
Weeks of school closure	-0.101***	0.070**	0.091**	0.077**	0.025
	(0.031)	(0.035)	(0.037)	(0.037)	(0.036)
Accommodations	-0.056	0.042	-0.143	-0.010	-0.004
	(0.087)	(0.110)	(0.095)	(0.110)	(0.080)
Panel m					
Weeks of school closure	-0.100***	0.069**	0.090**	0.077**	0.026
	(0.031)	(0.034)	(0.037)	(0.037)	(0.036)
Indoor sports	0.030	-0.038	-0.014	0.005	0.052
	(0.080)	(0.093)	(0.088)	(0.112)	(0.086)
Panel n					
Weeks of school closure	-0.101***	0.070**	0.091**	0.077**	0.025
	(0.031)	(0.035)	(0.037)	(0.037)	(0.036)
Outdoor sports	0.019	0.036	-0.080	-0.010	-0.003
	(0.071)	(0.085)	(0.080)	(0.090)	(0.070)

Notes: Each column in a panel is a separate regression. All outcome variables have mean 0 and sd 1. Panels show the results of estimating (4). Detailed descriptions on the pandemic measures can Materials and Methods. Which states have high restrictions on the respective pandemic measure can be found in Table S3. The number of observations for all regressions is 907. Standard errors are clustered at the state×school track×grade level. * $p < 0.1$, ** $p < 0.05$, *** $p < 0.01$. For more details see the paragraph on pandemic severity and stringency of pandemic measures.

Table S10: Heterogeneity analysis for adolescents' HRQoL and mental health

	KS-10	HBSC-SCL	SDQ	CES-DC	SCARED
Panel a: average effect by age					
11 y/o	-0.205***	0.211***	0.257***	0.155**	0.108*
	(0.061)	(0.067)	(0.057)	(0.066)	(0.065)
12 y/o	-0.159***	0.142***	0.177***	0.116*	0.067
	(0.050)	(0.054)	(0.049)	(0.061)	(0.054)
13 y/o	-0.144***	0.127***	0.137***	0.120***	0.056
	(0.039)	(0.042)	(0.039)	(0.045)	(0.041)
14 y/o	-0.113***	0.090**	0.116***	0.097**	0.036
	(0.033)	(0.035)	(0.037)	(0.040)	(0.037)
15 y/o	-0.085***	0.033	0.062	0.053	0.013
	(0.029)	(0.033)	(0.040)	(0.040)	(0.036)
16 y/o	-0.063*	0.012	0.010	0.039	-0.008
	(0.035)	(0.040)	(0.049)	(0.049)	(0.044)
17 y/o	-0.041	-0.018	-0.024	0.027	-0.016
	(0.046)	(0.045)	(0.059)	(0.061)	(0.057)
Panel b: average effect by sex					
Boys	-0.154***	0.094**	0.124***	0.111**	0.078*
	(0.036)	(0.042)	(0.044)	(0.047)	(0.043)
Girls	-0.063*	0.050	0.056	0.046	-0.016
	(0.037)	(0.039)	(0.041)	(0.042)	(0.043)
p value	0.024	0.323	0.069	0.196	0.060
Panel c: average effect by living space per child					
< median	-0.175***	0.098**	0.109**	0.114**	0.053
	(0.039)	(0.043)	(0.048)	(0.048)	(0.043)
≥ median	-0.046	0.046	0.062	0.042	0.002
	(0.033)	(0.040)	(0.039)	(0.042)	(0.037)
p value	0.002	0.283	0.301	0.171	0.231

Notes: Each column in a panel is a separate regression. All outcome variables have mean 0 and sd 1. All regressions include control variables for age and sex (except Panel b). In addition, regressions in Panel c include a dummy for above median living space per child. The number of observations for all regressions is 907. Standard errors are clustered at the state×school track×grade level. * $p < 0.1$, ** $p < 0.05$, *** $p < 0.01$. For more details see the paragraph on subgroup analysis.

Table S11: Results for parents' mental health and family climate

	PHQ-8	FC
Panel a: average effect		
Weeks of school closure	0.007	-0.140***
	(0.042)	(0.038)
Panel b: average effect by child's age		
11 y/o	0.118	-0.234***
	(0.074)	(0.069)
12 y/o	0.060	-0.159***
	(0.061)	(0.056)
13 y/o	0.036	-0.127***
	(0.050)	(0.043)
14 y/o	0.029	-0.140***
	(0.044)	(0.038)
15 y/o	0.005	-0.135***
	(0.042)	(0.037)
16 y/o	-0.053	-0.113**
	(0.048)	(0.048)
17 y/o	-0.102*	-0.066
	(0.060)	(0.064)
Panel c: average effect by child's sex		
Boys	0.054	-0.169***
	(0.051)	(0.047)
Girls	-0.038	-0.112**
	(0.047)	(0.044)
p value	0.074	0.252
Panel d: average effect by living space per child		
< median	-0.014	-0.203***
	(0.054)	(0.047)
≥ median	0.008	-0.084**
	(0.042)	(0.042)
p value	0.655	0.014

Notes: Shows the short-run impact of school closure on parental screening devices for depressive disorder (PHQ-8) and family climate (FC). Outcomes are parent-reported and have mean 0 and sd 1. For PHQ-8 higher values express more health problems. For FC higher values express better family climate. Each panel includes a separate regression of equation (1). Standard errors clustered at the state×school track×grade level. * $p < 0.1$, ** $p < 0.05$, *** $p < 0.01$ For more details see the paragraph on sensitivity analysis.

Table S12: Results for the change in call volume by prolonged school closure

	≤ 18/May	> 18/May	p value
30/Dec - 12/Jan	-0.406**	-0.365	0.8990
	(0.036)	(0.068)	
13/Jan - 26/Jan	-0.239	-0.172	0.8802
	(0.067)	(0.129)	
27/Jan - 09/Feb	-0.205	0.111	0.4291
	(0.072)	(0.087)	
10/Feb - 23/Feb	-0.051	-0.168	0.7789
	(0.067)	(0.109)	
24/Feb - 08/Mar	-0.303	-0.062	0.6111
	(0.068)	(0.156)	
09/Mar - 22/Mar	-0.479*	-0.307	0.6500
	(0.063)	(0.081)	
23/Mar - 05/Apr	0.076	0.168	0.7834
	(0.056)	(0.056)	
06/Apr - 19/Apr	0.066	0.295	0.4656
	(0.056)	(0.043)	
20/Apr - 03/May	0.315	0.751***	0.1792
	(0.056)	(0.049)	
04/May - 17/May	0.167	1.008***	0.0278
	(0.078)	(0.068)	
18/May - 31/May	0.134	0.832***	0.0310
	(0.059)	(0.046)	
01/Jun - 14/Jun	0.089	0.153	0.8349
	(0.048)	(0.043)	
15/Jun - 28/Jun	-0.172	0.798***	0.0093
	(0.062)	(0.077)	
29/Jun - 12/Jul	-0.219	0.815***	0.0011
	(0.059)	(0.041)	
13/Jul - 26/Jul	-0.138	0.148	0.5077
	(0.070)	(0.117)	
27/Jul - 09/Aug	-0.325	0.156	0.1293
	(0.045)	(0.056)	
10/Aug - 23/Aug	-0.415**	0.274	0.0872
	(0.045)	(0.117)	
24/Aug - 06/Sep	-0.258	0.167	0.2434
	(0.057)	(0.076)	

Continued on next page

Table S12 (cont.): Estimated change in call volume by school closure

	≤ 18/May	> 18/May	p value
07/Sep - 20/Sep	0.005	0.305	0.5057
	(0.035)	(0.167)	
21/Sep - 04/Oct	-0.536**	0.205	0.0400
	(0.044)	(0.086)	
05/Oct - 18/Oct	-0.277	0.319	0.0635
	(0.031)	(0.072)	
19/Oct - 01/Nov	-0.232	0.260	0.1915
	(0.033)	(0.109)	

Notes: Each column is a separate estimation of equation (9) and shows the estimated β_i. The column p value shows the p value of a test of equality of coefficients. Number of observations is 730 for each estimation. Standard errors are in parentheses. $^{*}\ p < 0.1$, $^{**}\ p < 0.05$, $^{***}\ p < 0.01$

For more details see the paragraph on the helpline analysis.

REFERENCES AND NOTES

1. American Academy of Pediatrics, American Academy of Child and Adolescent Psychiatry and Children's Hospital Association, AAP-AACAP-CHA Declaration of a National Emergency in Child and Adolescent Mental Health, 19 October 2021.

2. E. A. Holmes, R. C. O'Connor, V. H. Perry, I. Tracey, S. Wessely, L. Arseneault, C. Ballard, H. Christensen, R. Cohen Silver, I. Everall, T. Ford, A. John, T. Kabir, K. King, I. Madan, S. Michie, A. K. Przybylski, R. Shafran, A. Sweeney, C. M. Worthman, L. Yardley, K. Cowan, C. Cope, M. Hotopf, E. Bullmore, Multidisciplinary research priorities for the COVID-19 pandemic: A call for action for mental health science. *Lancet Psychiatry* **7**, 547–560 (2020).

3. Office of the Surgeon General. Protecting Youth Mental Health: The U.S. Surgeon General's Advisory (2021).

4. J. Lee, Mental health effects of school closures during COVID-19. *Lancet Child Adolesc. Health* **4**, 421 (2020).

5. M. Brülhart, V. Klotzbücher, R. Lalive, S. K. Reich, Mental health concerns during the COVID-19 pandemic as revealed by helpline calls. *Nature* **600**, 121–126 (2021).

6. M. Pierce, H. Hope, T. Ford, S. Hatch, M. Hotopf, A. John, E. Kontopantelis, R. Webb, S. Wessely, S. McManus, K. M. Abel, Mental health before and during the COVID-19 pandemic: A longitudinal probability sample survey of the UK population. *Lancet Psychiatry* **7**, 883–892 (2020).

7. T. Tanaka, S. Okamoto, Increase in suicide following an initial decline during the COVID-19 pandemic in Japan. *Nat. Hum. Behav.* **5**, 229–238 (2021).

8. R. Ghosh, M. J. Dubey, S. Chatterjee, S. Dubey, Impact of COVID -19 on children: Special focus on the psychosocial aspect. *Minerva Pediatr.* **72**, 226–235 (2020).

9. G. Wang, Y. Zhang, J. Zhao, J. Zhang, F. Jiang, Mitigate the effects of home confinement on children during the COVID-19 outbreak. *Lancet* **395**, 945–947 (2020).

10. A. Bagattini, Children's well-being and vulnerability. *Ethics Soc. Welf.* **13**, 211–215 (2019).

11. B. B. Brown, Adolescents' relationships with peers, *Handbook of Adolescent Psychology* (John Wiley & Sons, 2nd ed., 2004), pp 363–394.

12. I. H. A. De Goede, S. J. T. Branje, W. H. J. Meeus, Developmental changes in adolescents' perceptions of relationships with their parents. *J. Youth Adolesc.* **38**, 75–88 (2009).

13. N. Racine, B. A. McArthur, J. E. Cooke, R. Eirich, J. Zhu, S. Madigan. Global prevalence of depressive and anxiety symptoms in children and adolescents during COVID-19: A meta-analysis. *JAMA Pediatr.* **175**, 1142–1150 (2021).

14. Young people's mental health is finally getting the attention it needs. *Nature* **598**, 235–236 (2021).

15. K. Kishida, M. Tsuda, P. Waite, C. Creswell, S. Ishikawa, Relationships between local school closures due to the COVID-19 and mental health problems of children, adolescents, and parents in Japan. *Psychiatry Res.* **306**, 114276 (2021).

16. S. Tang, M. Xiang, T. Cheung, Y.-T. Xiang, Mental health and its correlates among children and adolescents during COVID-19 school closure: The importance of parent-child discussion. *J. Affect. Disord.* **279**, 353–360 (2021).

17. R. Viner, S. Russell, R. Saulle, H. Croker, C. Stansfield, J. Packer, D. Nicholls, A.L. Goddings, C. Bonell, L. Hudson, S. Hope, J. Ward, N. Schwalbe, A. Morgan, S. Minozzi, School closures during social lockdown and mental health, health behaviors, and well-being among children and adolescents during the first COVID-19 wave: A systematic review. *JAMA Pediatr.* **176**, 400–409 (2022).

18. H. Svaleryd, E. Björkegren, J. Vlachos, The impact of the COVID-19 school closure on adolescents' use of mental healthcare services in Sweden. medRxiv:2021.12.12.21267684 (2021).

19. U. Ravens-Sieberer, A. Kaman, M. Erhart, C. Otto, J. Devine, C. Löffler, K. Hurrelmann, M. Bullinger, C. Barkmann, N. A. Siegel, A. M. Simon, L. H. Wieler, R. Schlack, H. Hölling, Quality of life and mental health in children and adolescents during the first year of the COVID-19 pandemic: Results of a two-wave nationwide population-based study. *European Child & Adolescent Psychiatry*, 1–14 (2021).

20. F. Klasen, F. Reiß, C. Otto, A.-C. Haller, A.-K. Meyrose, D. Barthel, U. Ravens- Sieberer. Die

BELLA-Studie – das Modul zur psychischen Gesundheit in KiGGS Welle 2 (2017).

21. The Committee for the Prize in Economic Sciences in Memory of Alfred Nobel. Answering causal questions using observational data (2021)

22. J. Vornberger, Structural evidence on school closures in Germany during COVID-19 pandemic, Mimeo (2020).

23. UNESCO. Global monitoring of school closures caused by COVID-19, 12 (2021).

24. M. L. Hemmeter, M. Ostrosky, L. Fox, Social and emotional foundations for early learning: A conceptual model for intervention. *School Psych. Rev.* **35**, 583–601 (2006).

25. E. Croda, S. Grossbard, Women pay the price of COVID-19 more than men. *Rev. Econ. Househ.* **19**, 1–9 (2021).

26. M. Huebener, S. Waights, C. K. Spiess, N. A. Siegel, G. G. Wagner, Parental well-being in times of COVID-19 in Germany. *Rev. Econ. Househ.* **19**, 91–122 (2021).

27. P. Jäger, N. Ott, A. Brand, K. Fereidooni, Integration of newly arrived refugee children into the German school system. *Int. J. Environ. Res. Public Health* **18**, 7854 (2021).

28. J. M. Wooldridge. Two-way fixed effects, the two-way Mundlak regression, and difference- in-differences estimators. *SSRN Electron. J.* (2021).

29. J. D. Angrist, J.-S. Pischke, *Mostly Harmless Econometrics: An Empiricist's Companion*, Number 8769 in Economics Books (Princeton Univ. Press, 2009).

30. Institut für angewandte Sozialwissenschaft, infas 360 GmbH, and Institut für Hygiene und Public Health. Corona Daten Plattform.

31. B.-M. Kurth, Das RKI-Gesundheitsmonitoring – was es enthält und wie es genutzt werden kann. *Public Health Forum* **20**, 4.e1–4.e3.

32. U. Ravens-Sieberer, C. Otto, L. Kriston, A. Rothenberger, M. Döpfner, B. Herpertz-Dahlmann, C.

Barkmann, G. Schön, H. Hölling, M. Schulte-Markwort, F. Klasen; BELLA study group, The longitudinal BELLA study: Design, methods and first results on the course of mental health problems. *Eur. Child Adolesc. Psychiatry* **24**, 651–663 (2015).

33. C. Otto, F. Reiss, C. Voss, A. Wüstner, A.-K. Meyrose, H. Hölling, U. Ravens-Sieberer, Mental health and well-being from childhood to adulthood: Design, methods and results of the 11-year follow-up of the BELLA study. *Eur. Child Adolesc. Psychiatry* **30**, 1559–1577 (2021).

34. U. Ravens-Sieberer, A. Kaman, M. Erhart, J. Devine, R. Schlack, C. Otto, Impact of the COVID-19 pandemic on quality of life and mental health in children and adolescents in Germany. *Eur. Child Adolesc. Psychiatry*, **31**, 879–889 (2022).

35. U. Ravens-Sieberer, KIDSCREEN Group Europe. *The KIDSCREEN Questionnaires: Quality of Life Questionnaires For Children And Adolescents; Handbook* (Pabst Science Publishers, 2006).

36. S. Haugland, B. Wold, J. Stevenson, L. E. Aaroe, B. Woynarowska, Subjective health complaints in adolescence: A cross-national comparison of prevalence and dimensionality. *Eur. J. Public Health* **11**, 4–10 (2001).

37. R. Goodman. The strengths and difficulties questionnaire: A research note. *J. Child Psychol. Psychiatry* **38**, 581–586 (1997).

38. C. Barkmann, M. Erhart, M. Schulte-Markwort; BELLA Study Group, The German version of the centre for epidemiological studies depression scale for children: Psychometric evaluation in a population-based survey of 7 to 17 years old children and adolescents–Results of the BELLA study. *Eur. Child Adolesc. Psychiatry* **17**, 116–124 (2008).

39. B. Birmaher, D. A. Brent, L. Chiappetta, J. Bridge, S. Monga, M. Baugher, Psychometric properties of the screen for child anxiety related emotional disorders (SCARED): A replication study. *J. Am. Acad. Child Adolesc. Psychiatry* **38**, 1230–1236 (1999).

40. K. Kroenke, T. W. Strine, R. L. Spitzer, J. B. W. Williams, J. T. Berry, A. H. Mokdad, The PHQ-8 as a measure of current depression in the general population. *J. Affect. Disord.* **114**, 163–173 (2009).

41. K. Schneewind, M. Beckmann, A. Hecht-Jackl, Familienklima-Skalen. Bericht 8.1 und 8.2. Institut für Psychologie–Persönlichkeitspsychologie und Psychodiagnostik (1985).

42. U. Ravens-Sieberer, A. Kaman, C. Otto, A. Adedeji, A.-K. Napp, M. Becker, U. Blanck-Stellmacher, C. Löffler, R. Schlack, H. Hölling, J. Devine, M. Erhart, K. Hurrelmann, Seelische Gesundheit und psychische Belastungen von Kindern und Jugendlichen in der ersten Welle der COVID-19- Pandemie–Ergebnisse der COPSY-Studie. *Bundesgesundheitsblatt-Gesundheitsforschung- Gesundheitsschutz* **64**, 1512–1521 (2021).

43. U. Ravens-Sieberer, M. Erhart, J. Devine, M. Gilbert, F. Reiss, C. Barkmann, N. A. Siegel, A. M. Simon, K. Hurrelmann, R. Schlack, H. Hölling, L. H. Wieler, A. Kaman, Child and adolescent mental health during the COVID-19 pandemic: Results of the three-wave longitudinal COPSY study. *J. Adolesc. Health* **71**, 570–578 (2022).

44. T. R. Coker, T. L. Cheng, M. Ybarra, Addressing the long-term effects of the COVID-19 pandemic on children and families: A report from the national academies of sciences, engineering, and medicine. *JAMA* **329**, 1055–1056 (2023).

45. M. Lange, R. Hoffmann, E. Mauz, R. Houben, A. Gößwald, A. S. Rosario, B. Kurth, KiGGS wave 2 longitudinal component – data collection design and develop- ments in the numbers of participants in the KiGGS cohort. **3**, 92–107 (2018).

46. K. R. Krause, S. Chung, A. O. Adewuya, A. M. Albano, R. Babins-Wagner, L. Birkinshaw, P. Brann, C. Creswell, K. Delaney, B. Falissard, C. B. Forrest, J. L. Hudson, S. Ishikawa, M. Khatwani, C. Kieling, J. Krause, K. Malik, V. Martínez, F. Mughal, T. H. Ollendick, S. H. Ong, G. C. Patton, U. Ravens-Sieberer, P. Szatmari, E. Thomas, L. Walters, B. Young, Y. Zhao, M. Wolpert, International consensus on a standard set of outcome measures for child and youth anxiety, depression, obsessive-compulsive disorder, and post-traumatic stress disorder. *Lancet Psychiatry* **8**, 76–86 (2021).

47. U. Ravens-Sieberer, M. Herdman, J. Devine, C. Otto, M. Bullinger, M. Rose, F. Klasen, The European KIDSCREEN approach to measure quality of life and well-being in children: Development, current application, and future advances. *Qual. Life Res.* **23**, 791–803 (2014).

48. L. Sawyer Radloff, The CES-D Scale: A self-report depression scale for research in the general population. *Appl. Psychol. Measur.* **1**, 385–401 (1977).

49. K. Weitkamp, G. Romer, S. Rosenthal, S. Wiegand-Grefe, J. Daniels, German screen for child anxiety related emotional disorders (SCARED): Reliability, validity, and cross-informant agreement in a clinical sample. *Child Adolesc. Psychiatry Ment. Health* **4**, 19 (2010).

50. Nummer gegen Kummer (2021); www.nummergegenkummer.de.

51. S. Correia, A feasible estimator for linear models with multi-way fixed effects (2016); http://scorreia.com/research/hdfe.pdf.

52. A. Abadie, S. Athey, G. W. Imbens, J. M. Wooldridge, When should you adjust standard errors for clustering? *Q. J. Econ.* **138**, 1–35 (2022).

53. C. Eiser, R. Morse. Can parents rate their child's health-related quality of life? Results of a systematic review. *Qual. Life Res.* **10**, 347–357 (2001).

54. P. Upton, J. Lawford, C. Eiser, Parent-child agreement across child health-related quality of life instruments: A review of the literature. *Qual. Life Res.* **17**, 895–913 (2008).

55. T. Hale, N. Angrist, R. Goldszmidt, B. Kira, A. Petherick, T. Phillips, S. Webster, E. Cameron-Blake, L. Hallas, S. Majumdar, H. Tatlow, A global panel database of pandemic policies (Oxford COVID-19 Government Response Tracker). *Nat. Hum. Behav.* **5**, 529–538 (2021).

56. E. Mathieu, H. Ritchie, L. RodÅLes-Guirao, C. Appel, D. Gavrilov, C. Giattino, J. Hasell, B. Macdonald, S. Dattani, D. Beltekian, E. Ortiz-Ospina, M. Roser. Data on COVID-19 (Coronavirus), Our World in Data (2023).

57. Institut für angewandte Sozialwissenschaft, infas 360 GmbH, and Institut für Hygiene und Public Health. Corona Daten Plattform: Infektionen Kreise.

58. Institut für angewandte Sozialwissenschaft, infas 360 GmbH, and Institut für Hygiene und Public Health. Corona Daten-Plattform: Todesfälle.
